# Dynamic covalent synthesis of aryleneethynylene cages through alkyne metathesis: dimer, tetramer, or interlocked complex?†
†Electronic supplementary information (ESI) available: Materials and the general methods, synthetic procedures, computational method, and characterization data of selected compounds. See DOI: 10.1039/c5sc04977f


**DOI:** 10.1039/c5sc04977f

**Published:** 2016-02-12

**Authors:** Qi Wang, Chao Yu, Chenxi Zhang, Hai Long, Setareh Azarnoush, Yinghua Jin, Wei Zhang

**Affiliations:** a Department of Chemistry and Biochemistry , University of Colorado , Boulder , Colorado 80309 , USA . Email: yinghua.jin@colorado.edu ; Email: wei.zhang@colorado.edu; b National Renewable Energy Laboratory , Golden , Colorado 80401 , USA

## Abstract

A dynamic covalent approach towards rigid aryleneethynylene covalent organic polyhedrons (COPs) was explored. Our study on the relationship of the COP structures and the geometry of their building blocks reveals that the topology of aryleneethynylene COPs strongly depends on the size of the building blocks. A tetramer (*D*_2h_ symmetric), dimer, or interlocked complex can be formed from monomers with the same face-to-edge angle but in different sizes. As alkyne metathesis is a self-exchange reaction and non-directional, the cyclooligomerization of multi-alkyne monomers involves both intramolecular cyclization and intermolecular metathesis reaction, resulting in complicated thermodynamic process disturbed by kinetic competition. Although a tetrahedron-shaped tetramer (*T*_d_ symmetric) has comparable thermodynamic stability to a *D*_2h_ symmetric tetramer, its formation is kinetically disfavored and was not observed experimentally. Aryleneethynylene COPs consist of purely unsaturated carbon backbones and exhibit large internal cavities, which would have interesting applications in host–guest chemistry and development of porous materials.

## Abstract 2

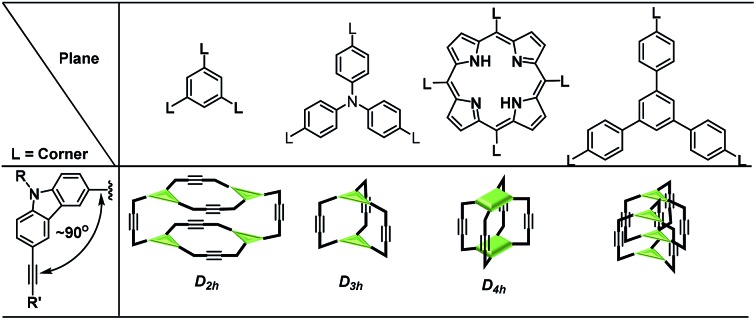
A modular dynamic covalent approach towards rigid aryleneethynylene covalent organic polyhedrons (COPs) and the mechanistic features were explored.

## Introduction

Dynamic covalent chemistry (DC_v_C) based on reversible covalent bonding provides a powerful platform for efficient assembly of purely organic, complex molecular architectures from simple building blocks.^1^–^4^ Such a thermodynamically-controlled assembly process, coupled with template assistance in certain cases, has presented striking illustrations of elaborate molecular topologies,^5^–^7^ such as Borromean rings,^8^ Solomon knots,^9^ or trefoil knot.^10^ The high efficiency of such molecular assembly is governed by the reversibility of the bond formation, the equilibrium thermodynamics, and the structures of building blocks, which are programmed in such a way to fit together and predominantly produce a single species at the equilibrium.^6^,^11^–^13^ The well-known reversible covalent chemistry with suitable kinetics of bond cleavage and formation includes imine formation,^14^–^17^ boronic acid condensation,^13^,^18^ and disulfide exchange reactions.^12^,^19^ With the recent advent of various catalysts,^20^–^25^ alkyne metathesis has rapidly emerged as a viable dynamic covalent reaction, which provides robust and linear ethynylene linkages. One-step assembly of shape-persistent macrocycles *via* alkyne metathesis has been well demonstrated in the past two decades.^26^–^30^ However, the assembly of more challenging three-dimensional molecular cages through alkyne metathesis has rarely been explored.

Molecular cages have shown great potential in molecular recognition, chemical sensing, catalysis, and gas adsorption/separation.^31^–^35^ Self-assembly of supramolecular cages^36^–^40^ *via* metal coordination or hydrogen bonding has provided the foundation for advances in covalent assembly of purely organic molecular cages, also called covalent organic polyhedrons (COPs).^11^,^15^,^41^ The major advantages of using alkyne metathesis in assembly of COPs include high rigidity and linearity of ethynylene bonds, which can provide fully conjugated shape-persistent organic scaffolds. The shape-persistency of aryleneethynylene molecular cages renders non-collapsible, well-defined internal cavity, which can host a variety of intriguing guest molecules (*e.g.* fullerenes). In addition, ethynylene linked COPs exhibit much higher stability, compared to those linked by C=N bonds or B–O bonds, which are usually labile and prone to hydrolysis. Ethynylene-linked COPs have generally been prepared through kinetically-controlled Sonogashira or Glaser-type coupling.^42^–^47^ However, such synthetic methods lack selectivity, high yields, and efficiency and thus limit the wide applications of these rigid cage molecules. Herein, we report the covalent assembly of aryleneethynylene COPs through one-step alkyne metathesis starting from readily accessible precursors. We studied the relationship of the COP structures and the geometry of their building blocks, and investigated the possible pathway to various aryleneethynylene COPs. As alkyne metathesis is a self-exchange reaction and non-directional, it distinguishes itself from commonly-practiced directional dynamic bonds, including hydrogen bonds, metal–ligand dative bonds, and imine bonds. Such a peculiar feature requires unique building block designs and adds additional complexity by introducing kinetic factors to thermodynamically controlled process, which has rarely been discussed previously.^48^,^49^ We demonstrate the dimensions of building blocks and kinetic competitions play critical roles in determining the topology of the assembled structure through alkyne metathesis.

## Results and discussion

### Synthesis of COPs

**Fig. 1:**
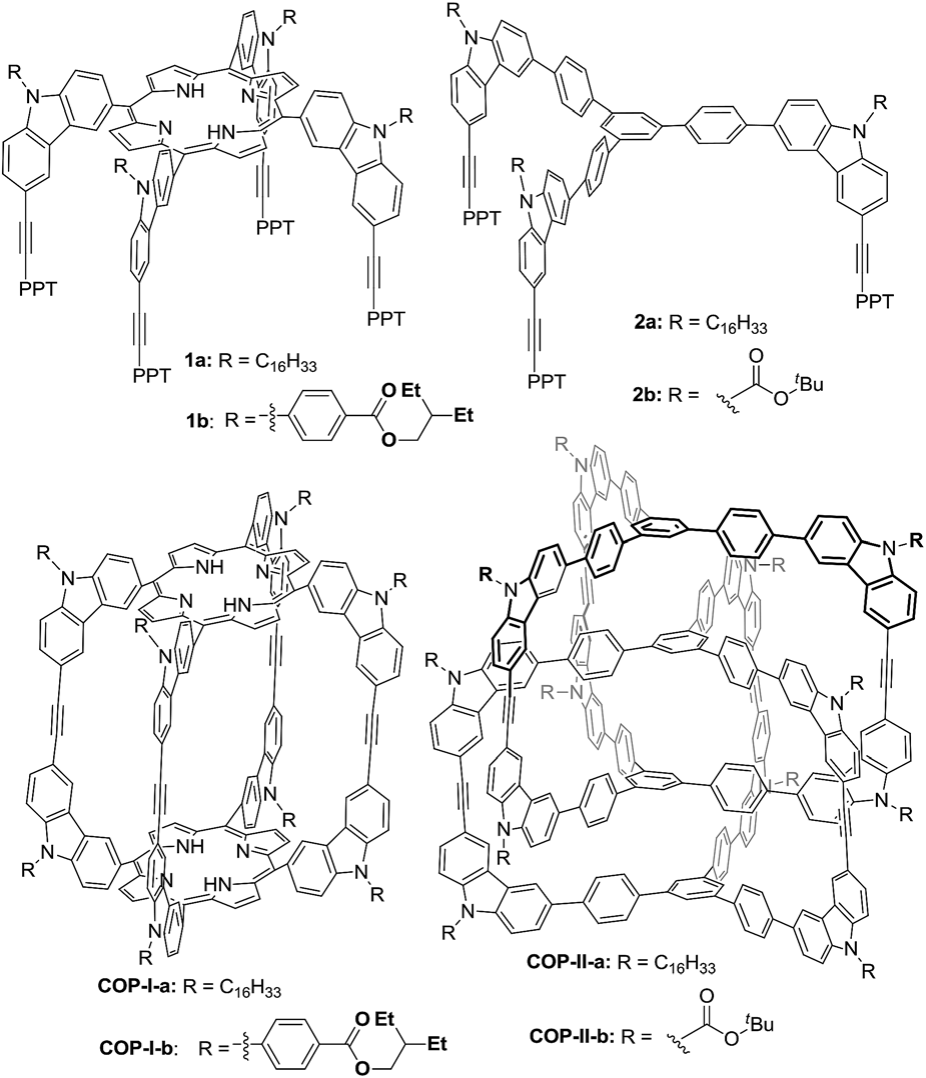
Structures of building blocks and COPs.

**Scheme 1:**
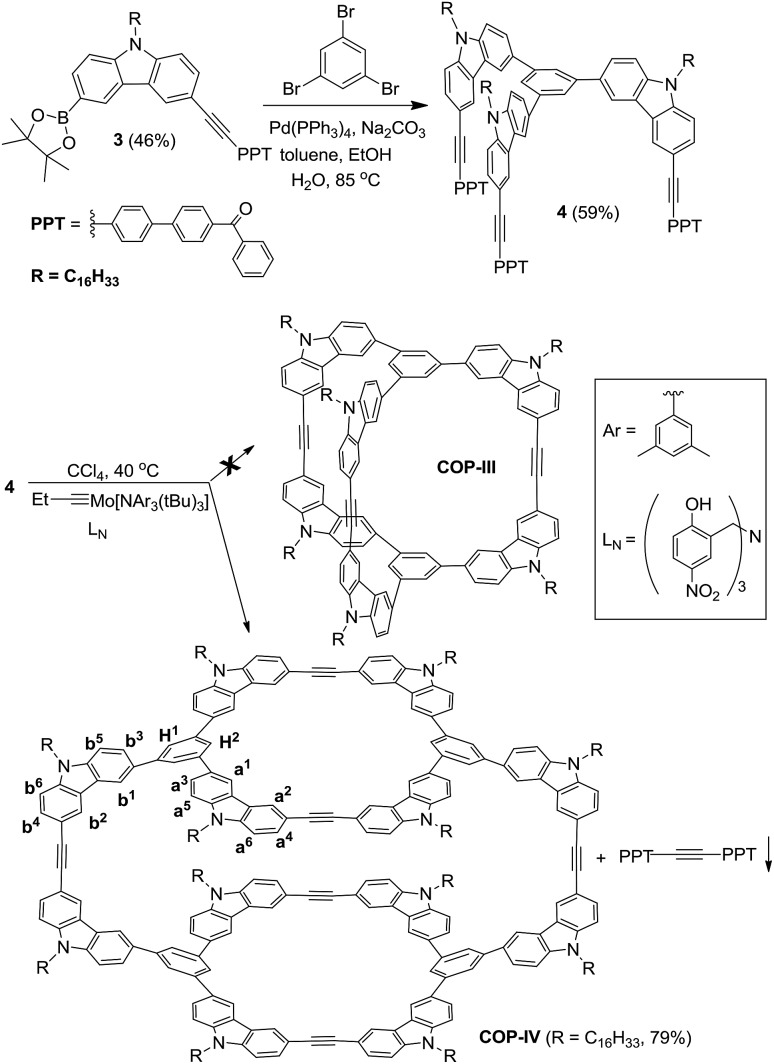
Synthesis of monomer **4** and **COP-IV**.

Previously, we reported covalent assemblies of dimeric **COP-I** and interlocked dimer complex (**COP-II**) through one-step alkyne metathesis cyclooligomerization of simple building blocks (**1**, and **2**) (Fig. 1).^50^,^51^ Both of the building blocks consist of one panel (face) and multiple identical carbazole arms (edge), which are symmetrically placed on the periphery of the panel with the face-to-edge angle close to 90°. While monomer **1** provides high yielding of **COP-I** (72%) without any noticeable amount of interlocked species, monomer **2** predominantly forms interlocked complex **COP-II** (59%) along with a small amount of single dimer (6%). Intrigued by these results, we replaced the central panel of the monomers with a benzene ring, the smallest possible planar moiety available, wondering what would be the assembly product, interlocked complex or a dimer cage **COP-III**. The synthesis of tricarbazolyl-substituted benzene monomer **4** is straightforward (Scheme 1). (Benzoyldiphenyl)acetylene (PPT) was attached as the end group in order to precipitate the reaction byproduct (PPT—≡—PPT) and drive the metathesis equilibrium to completion. The alkyne metathesis of the monomer **4** was completed within 2 h under the catalysis of molybdenum(vi) carbyne complex prepared from molybdenum(vi) trisamide precursor EtC≡Mo[N(*t*-Bu)Ar]_3_ and triphenolamine ligand (L_N_).^52^,^53^ The product was isolated in good yield (79%) through column chromatography purification.

**Scheme 2:**
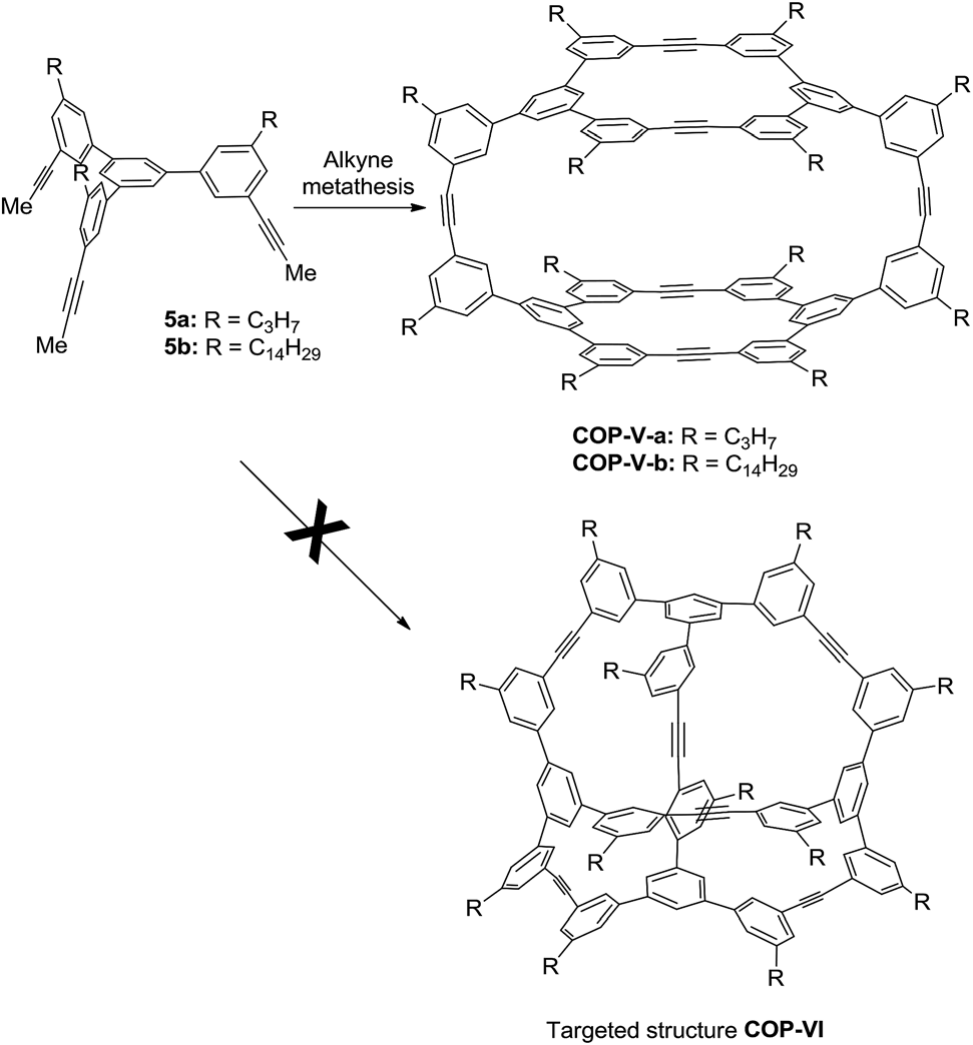
Alkyne metathesis of monomer **5**.

The product was then characterized by GPC, MALDI-MS and NMR spectroscopy. GPC trace of the product shows one sharp peak with polydispersity index of 1.04, supporting the formation of a single species (Fig. S8†). MALDI-MS of the product shows a single peak with *m*/*z* corresponding to a species containing four monomer units and we were unable to detect any noticeable amount of the expected dimer **COP-III**. ^1^H NMR spectrum of the product shows two sets of sharp proton signals in both aromatic and aliphatic regions with the ratio close to 2 : 1, which do not undergo coalescence or sharpening at elevated temperature (59 °C). These ^1^H NMR patterns are highly similar to the *D*_2h_ symmetric tetrameric cage (**COP-V**), whose structure has been unambiguously determined by single crystal X-ray analysis (Scheme 2).^54^ We therefore assigned the product to **COP-IV**, in which the two “bridging” arms are in different chemical environment from those forming macrocycles, leading to the splitting of the originally identical protons into two groups. The assignment was further confirmed by various 2D NMR experiments, gCOSY, ROSEY, NOESY, HSQC and HMBC.

**Scheme 3:**
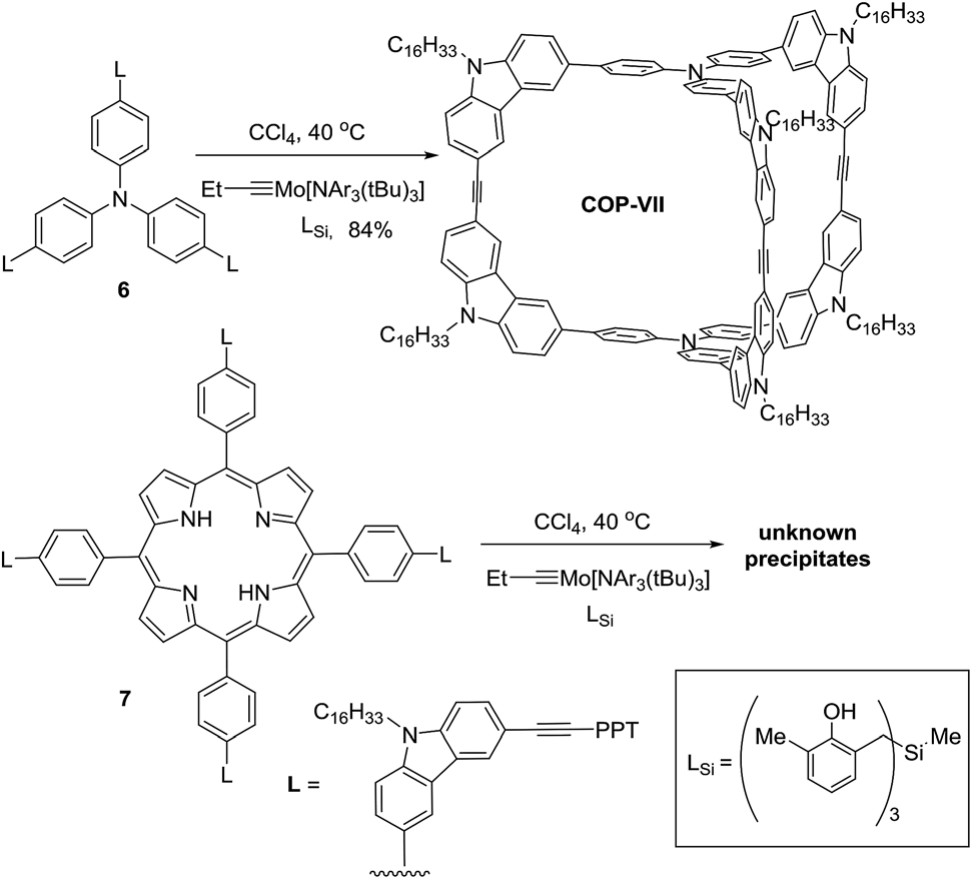
Alkyne metathesis of monomers **6** and **7**.

It is unexpected to obtain *D*_2h_-symmetric tetrameric cages from both tritopic monomers **4** and **5**, whose face-to-edge angles are 90° and 60°, respectively. Intrigued by these results, we studied two more examples using similar tritopic compound **6** and tetratopic compound **7**, whose central panels are progressively enlarged from benzene but with locked face-to-edge angles of 90°. The alkyne metathesis was performed using molybdenum carbyne catalyst consisting of triphenol silane ligand (L_Si_).^55^ Interestingly, monomer **6** produces symmetrical dimer **COP-VII** instead of a *D*_2h_ symmetric tetramer in excellent isolated yield (84%), whereas **7** produces unknown precipitates with a trace amount of a dimer species, which was only observed in the MALDI-MS spectrum of the crude product mixture (Scheme 3). In both cases, long alkyl chains (C_16_H_33_) are attached to the monomer in order to prevent the premature precipitation of the oligomeric/polymeric products from the reaction medium. Many attempts under various conditions (temperature, solvents, catalysts loading, high dilution) failed to provide a soluble discrete species from monomer **7**.

### Alkyne metathesis: catalysts, solubilizing groups, and equilibrium

We used molybdenum carbyne catalytic system containing multidentate ligand in the alkyne metathesis cyclooligomerization approach. The catalyst solution was prepared *in situ* by mixing Mo(vi) carbyne precursor Et≡Mo[NAr(*t*Bu)]_3_ with multidentate triphenolsilane (L_Si_) or triphenolamine (L_N_) ligands. Both catalytic systems are highly active and have long lifetime, thus suitable for the cage synthesis.

Alkyl substituents are attached to prevent precipitation of the reaction intermediates from the solution. In order to reach the equilibrium with the predominant formation of the desired cage products, precipitation (kinetic traps) should be avoided, which cannot further participate in the dynamic equilibrium process. We tested various alkyl chains, linear or branched, long or short. We found alkyl substituents have little effect on the control of cage topologies, that being said, their main contribution is to maintain good solubility of reaction intermediates.

**Scheme 4:**
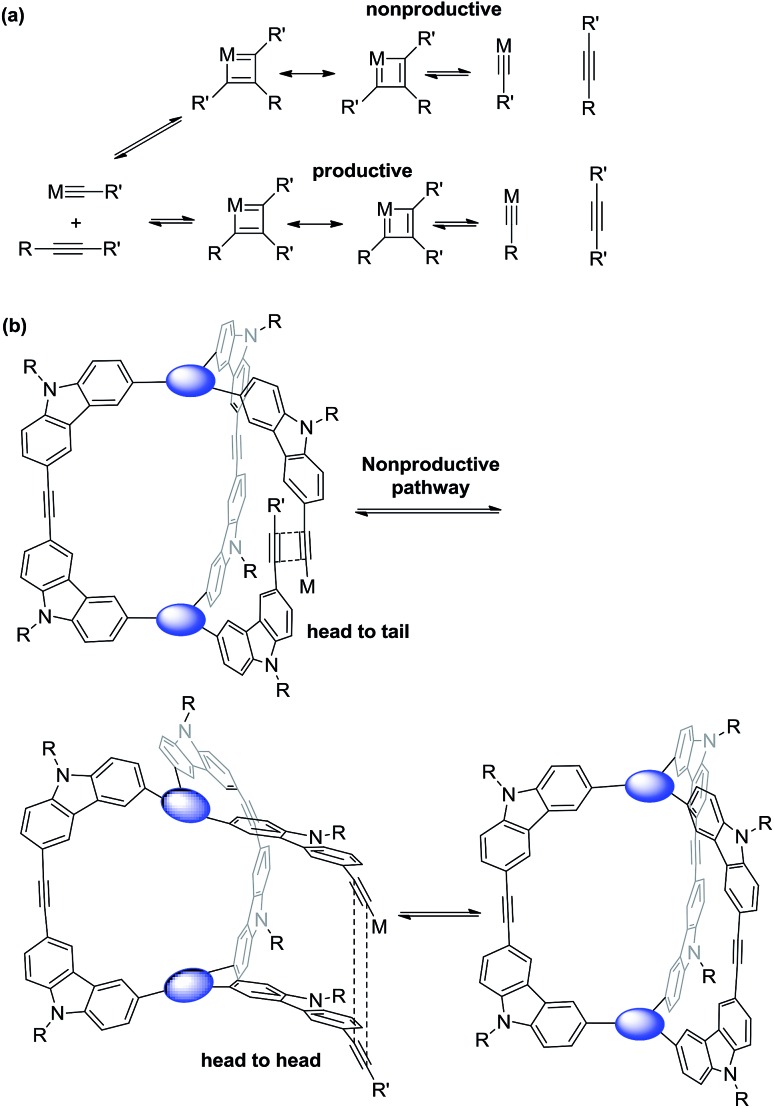
(a) General alkylidyne mechanism of alkyne metathesis; (b) head-to-tail or head-to-head arrangement of alkylidynes leading to nonproductive or productive pathway in the cage synthesis.

Alkyne metathesis is an exchange reaction involving redistribution of alkylidyne units between two alkynes through the proposed formation of metallacyclobutadienes followed by cycloreversion (Scheme 4a). Since alkyne metathesis involves reversible equilibrium, in order to achieve high conversion of monomers, one alkyne product is usually removed through scavenging it with molecular sieves, application of vacuum, or precipitation. In the cage synthesis, methyl substituted or benzoyldiphenyl substituted alkynes were used as end groups in order to scavenge small 2-butyne by molecular sieves or precipitate bis(benzoyldiphenyl)acetylene byproduct. Both approaches are able to produce the cage molecules successfully, each with advantages and disadvantages. The advantage of the former approach is the relatively easy synthesis, while the purification of multi-propyne substituted monomers is sometimes difficult due to the low polarity of highly carbon-rich aromatic monomers. On the other hand, benzoyldiphenylacetylene groups in the latter approach can increase polarity of the monomers and thus facilitate their purification. However, such approach is not atom economic, losing significant mass by precipitation, and the monomer preparation involves more synthetic steps.

### Geometry of building blocks and topology of cages

The size and shape complementarity of building blocks are usually considered to be critical for the assembly of supramolecules.^31^,^32^,^36^–^39^ In general, the angular disposition of connection sites of building blocks determines the topology, whereas the size of building blocks determines the physical dimension of the assembly product. As ethynylene bonds are linear, closely resembling metal–ligand dative bonds, a tenable view is that they share similar geometrical requirements for building blocks when employed in the assembly of discrete molecules. However, our study shows that the dynamic assembly involving alkyne metathesis is more complicated, in which the variation in building block sizes leads to a dramatic change in not only the size but also the topology of the molecular assemblies. When the central panel was varied from porphyrin, to 1,3,5-triphenyl benzene, or to benzene, a simple dimer cage (**COP-I**), an interlocked complex consisting of two dimer cages (**COP-II**), or a tetrameric cage with *D*_2h_ symmetry (**COP-IV**) were obtained as the assembly products, respectively (Table 1). It should be noted that, in each aforementioned case, the face-to-edge angle was locked to be around ∼90° by using 3,6-disubstitued carbazole moieties as the angular corner pieces. Originally, we expected dimeric cages (*e.g.***COP-III**) similar to **COP-I** from all the monomers with face-to-edge angle of 90°. At first glance, such dimers would be entropically- and enthalphically-favored, since they consist of the fewest possible monomer units connected with minimum angle strain. Therefore, we were surprised to observe the predominant formation of tetrameric **COP-IV** with *D*_2h_ symmetry from monomer **4**. However, when we further scrutinized the reaction pathway, we found the possible steric reasons responsible for the disfavored formation of a dimer. As shown in Scheme 4a, depending on the regioselectivity of the addition of acetylene units to Mo≡C, there are two possible metathesis outcomes: nonproductive and productive pathways. In order to form a dimeric cage, in the final cyclization step, the two arms have to approach each other in head-to-head conformation to undergo a productive metathesis (Scheme 4b). If the arrangement of such a conformation involves significant angle strains and the two arms are unable to come in close contact, they likely seek another exchange partner to undergo intermolecular reaction to form a tetrameric cage. The steric restriction to undergo intramolecular alkyne metathesis cyclization is likely one of the main reasons for the preferred formation of **COP-IV** with *D*_2h_ symmetry rather than the initially targeted dimer **COP-III** when a small benzene ring is used as the central panel of the monomer. Presumably, larger porphyrin or triphenyl benzene moiety can provide more flexibility than a benzene ring and thus can effectively release the angle strain built up during the final cyclization step.

**Table 1:** Combination of various planar and angular moieties for assembly of COPs

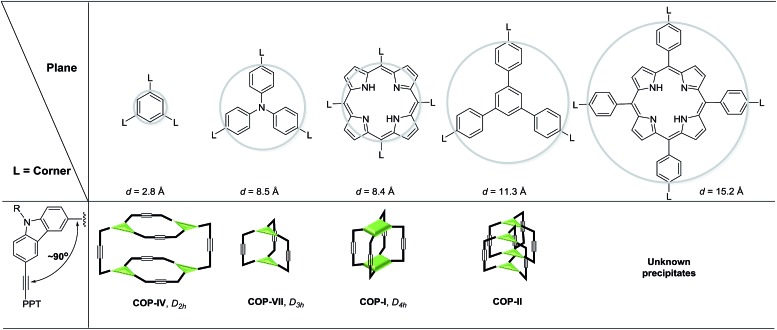

### Kinetic competition

The next puzzling observation that attracted our attention was the formation of *D*_2h_ symmetric tetramer **COP-V** from monomer **5** whose central panel is benzene and face-to-edge angle is ∼60°. Again, we failed to obtain the initially targeted tetrahedron-shaped cage **COP-VI**, which would have face-to-edge angle of ∼54.7°, closely matching the face-to-edge angle of monomer **5**. In order to evaluate the relative thermodynamic stabilities of **COP-V** and **COP-VI**, we examined their energy-minimized conformations and calculated the energies. The Amber 11.0 molecular dynamics program package was used to optimize the structures of cages by energy minimization for 1000 steps using the general Amber force field (GAFF)^56^ with the charge parameters computed by the AM1-BCC method.^57^ Interestingly, our computational calculations indicate that there is no significant energy gap between the tetramer **COP-V** and our original target, tetrahedron-shaped **COP-VI**.

**Fig. 2:**
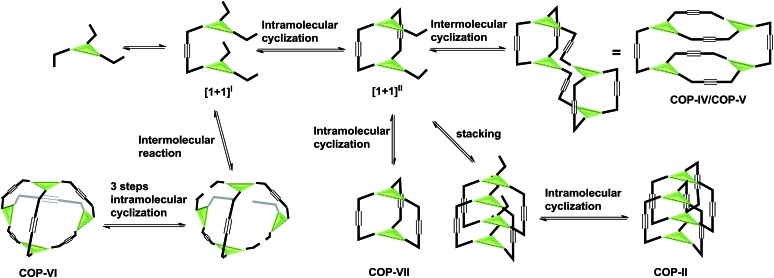
Possible reaction pathways to COPs.

Intrigued by these observations, we next investigated the possible kinetic competition between the formation of **COP-V** and **COP-VI**. It should be noted that the pendant arms (carbazole or benzene) attached to the central panels (porphyrin or benzene) can freely rotate in all the monomers tested and are not preorganized to form a particular product. Since monomers are multitopic and self-reacting, the initial formation of the dimeric intermediate **[1+1]^I^** likely triggers intramolecular cyclization to form macrocyclic intermediates **[1+1]^II^** and further to form cages (Fig. 2). We indeed observed the formation of a significant amount of macrocyclic dimers **[1+1]^II^** in the early stage of dynamic assembly of **COP-V**, experimentally supporting the above pathway.^54^ By contrast, the formation of tetrahedron-shaped **COP-VI**, in which each monomer unit has to connect with three other monomers, involves at least one more intermolecular reaction and three intramolecular cyclization steps from the dimeric intermediate **[1+1]^I^**. Therefore, the formation of dimeric cages (*e.g.***COP-I**, **COP-VII**), interlocked dimer complexes (**COP-II**) or a tetramer **COP-IV** or **COP-V** are kinetically preferred. Unless there is a strong thermodynamic driving force towards **COP-VI**, its formation could thus be limited. When a benzene ring is used as the planar central piece in a monomer, the pendant third arms on the macrocyclic intermediate **[1+1]^II^** prefer intermolecular cyclization to form a tetramer **COP-IV** or **COP-V** rather than forming a dimer cage, presumably due to the significant angle strain built up in the latter case as discussed previously. The assemblies of building blocks vary significantly with the choice of connecting chemical bonds that display different properties, such as kinetics, directional/non-directional, bond angles, and torsional/rotational freedoms. The dynamic assembly of COPs through alkyne metathesis, which is self-reacting, represents a complex system in which both thermodynamic and kinetic factors have to be considered.

## Conclusions

The design and syntheses of aryleneethynylene COPs through dynamic alkyne metathesis cyclooligomeriztion approach and the effect of the size and geometry of the building units on the final outcome of assembly process were systematically investigated. Alkyne metathesis is a non-directional exchange reaction, which requires regioselective arrangement of ethynylene groups in order to proceed the productive pathway. As a result, the dynamic assembly through alkyne metathesis is significantly influenced by the geometrical properties of building blocks. Our study indicates the size of building blocks plays a significant role in determining the topology of the assembly structure. Depending on the size of top and bottom panels, dimeric cage, tetrameric cage, or interlocked dimeric cage were obtained from the monomers with the same face-to-edge angle. The starting materials for the cage products are designed to be multitopic and self-reacting, which inevitably raise the kinetic competition between intramolecular and intermolecular cyclization. The initial bimolecular reaction to form **[1+1]^I^** dimer, followed by a series of subsequent intramolecular cyclizations rather than intermolecular reactions, would be preferred. Therefore, the formation of tetrahedron-shaped COPs (*e.g.***COP-VI**), which involves more intermolecular metathesis steps, is kinetically disfavored. However, formation of such a cage structure can be predominant when there is a strong thermodynamic preference. The dynamic assembly through non-directional alkyne metathesis is complicated, in which both thermodynamic stability and kinetic factors could play critical roles in determining the final product. Although a general predictable relationship between building blocks and the assembled structures can be deduced by exploiting analogy between supramolecular and covalent assembly, accurate assessment of such a relationship still remains challenging considering the diversity of dynamic reactions and the complexity of dynamic assembly process. We carefully design building blocks based on literature precedents and experience, however, all too often our planning instead brings something surprising, yet logical. Our study will shed some light on this intriguing dynamic covalent assembly, which has shown tremendous potential in chemistry, material science and biology.

## Supplementary Material

Supplementary information
