# Supplementary material for: Dynamic covalent synthesis of aryleneethynylene cages through alkyne metathesis: dimer, tetramer, or interlocked complex?
Source: Chem Sci. 2016 Feb 12;7(5):3370–6. doi: 10.1039/c5sc04977f (PMC6007092; doi:10.1039/c5sc04977f)
Supplement: Supplementary file 1 [file SC-007-C5SC04977F-s001.pdf]

Electronic Supplementary Material (ESI) for Chemical Science.
This journal is © The Royal Society of Chemistry 2016

Dynamic Covalent Synthesis of Aryleneethynylene Cages through Alkyne Metathesis: Dimer, Tetramer, or Interlocked Complex?

Qi Wang,[a†] Chao Yu,[a†] Chenxi Zhang,[a] Hai Long,[b] Setareh Azarnoush,[a] Yinghua Jin*[a] and Wei Zhang*[a]

[a]Department of Chemistry and Biochemistry, University of Colorado, Boulder, Colorado, 80309, USA and [b]National Renewable Energy Laboratory, Golden, Colorado 80401, USA

Table of Contents

Materials and general synthetic methods 2
Synthetic procedures 3
Kinetic study of COP formation 10
Computational method 10
NMR characterization of **COP-IV** 11
NMR spectra of new compounds 17
References 21

Materials and general synthetic methods

Reagents and solvents were purchased from commercial suppliers and used without further purification, unless otherwise indicated. Tetrahydrofuran (THF), toluene, CH_2Cl_2 and dimethylformamide (DMF) were purified by the MBRAUN solvent purification systems. Compounds **3**,[1] **S-1**,[2] and **S-2**[3] were prepared as previously reported.

All reactions were conducted under dry nitrogen in oven-dried glassware, unless otherwise specified. All the alkyne metathesis reactions were conducted in the glovebox. The solvents used in alkyne metathesis were dried over 4 Å molecular sieves. Solvents were evaporated using a rotary evaporator after workup. Unless otherwise specified, the purity of the compounds was ≥ 95 % based on ^{1}H NMR spectral integration.

Flash column chromatography was performed by using a 100–150 times weight excess of flash silica gel 32–63 μm from Dynamic Absorbants Inc. Fractions were analyzed by TLC using TLC silica gel F254 250 μm precoated-plates from Dynamic Absorbants Inc. Analytical gel permeation chromatography (GPC) was performed using a Viscotek GPCmaxTM, a Viscotek Model 3580 Differential Refractive Index (RI) Detector, a Viscotek Model 3210 UV/VIS Detector and a set of two Viscotek Viscogel columns (7.8 × 30 cm, l-MBLMW-3078, and l-MBMMW-3078 columns) with THF as the eluent at 30 °C. The analytical GPC was calibrated using monodisperse polystyrene standards.

MALDI-TOF Mass spectra were obtained on the Voyager-DE™ STR Biospectrometry Workstation using sinapic acid (SA) as the matrix. The high resolution mass spectra were obtained on Waters SYNAPT G2 High Definition Mass Spectrometry

System. Analyte molecules were diluted in ESI solvents (methanol, chloroform or acetonitrile/water mixture) to final concentrations of 10 ppm or lower. The solution was injected into the electrospray ionization (ESI) source at a rate of 5 μL/min. Either the ESI+ or ESI− mode was used in reference to the molecular properties. Accurate mass analysis was performed by using the Lock Mass calibration feature with the instrument.

NMR spectra were taken on Inova 400 and Inova 500 spectrometers. $CDCl_3$ (7.26 ppm), benzene-d_6 (7.16 ppm), toluene-d_8 (2.08 ppm) were used as internal references in ^{1}H NMR spectra, and $CDCl_3$ (77.16 ppm) was used in ^{13}C NMR spectra. ^{1}H NMR data were reported in order: chemical shift, multiplicity (s, singlet; d, doublet; t, triplet; q, quartet; m, multiplet), coupling constants (J, Hz), number of protons.

Synthetic procedures

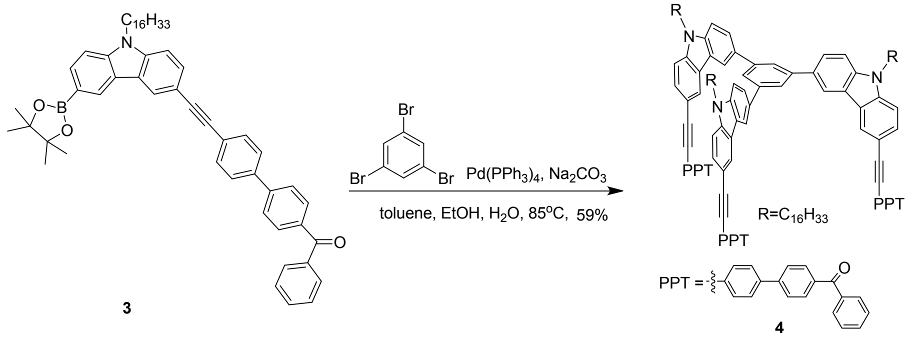

Synthesis of 4: To a Schlenk tube were added **3** (290 mg, 0.36 mmol), 1,3,5-tribromobenzene (27 mg, 0.087 mmol), Na_2CO_3 (83 mg, 0.78 mmol) and $Ph(PPh_3)_4$ (10 mg, 0.0086 mmol). Toluene (10 mL), H_2O (4 mL) and EtOH (4 mL) were then added and the mixture was heated at 85 °C for overnight. After the mixture was cooled to room temperature, it was washed with sat. NH_4Cl (50 mL). The aqueous layer was discarded. The organic layer was dried over Na_2SO_4 and the solvent was removed under reduced

pressure. The residue was purified by flash column chromatography (CH_2Cl_2/hexane, 1/1–4/1, *V*/*V*) to give the product (115 mg, 59%): ^{1}H NMR (500 MHz, $CDCl_3$) δ 8.56 (d, J = 1.5 Hz, 3H), 8.48 (d, J = 1.4 Hz, 3H), 8.07 (s, 3H), 7.98 (dd, J = 8.5, 1.6 Hz, 3H), 7.89 – 7.84 (m, 6H), 7.84 – 7.80 (m, 6H), 7.71 (dd, J = 8.4, 1.5 Hz, 3H), 7.70 – 7.64 (m, 12H), 7.63 – 7.59 (m, 9H), 7.56 (d, J = 8.6 Hz, 3H), 7.53 – 7.48 (m, 6H), 7.44 (d, J = 8.5 Hz, 3H), 4.36 (t, J = 7.0 Hz, 6H), 1.98 – 1.88 (m, 6H), 1.49 – 1.40 (m, 6H), 1.38-1.34 (m, 6H), 1.34 – 1.18 (m, 66H), 0.87 (t, J = 7.0 Hz, 9H); ^{13}C NMR (101 MHz, $CDCl_3$) δ 196.4, 144.6, 143.3, 140.9, 140.7, 139.1, 137.9, 136.5, 133.3, 132.6, 132.2, 131.0, 130.2, 129.7, 128.5, 127.4, 126.9, 126.2, 125.0, 124.6, 124.2, 123.3, 123.3, 119.5, 113.5, 109.6, 109.2, 92.6, 87.7, 43.7, 32.1, 29.9, 29.8, 29.8, 29.7, 29.6, 29.3, 27.6, 22.9, 14.4. MALDI-TOF (*m*/*z*): $[M+H]^+$ calcd. for $C_{153}H_{159}O_3N_3$, 2087.24; found: 2088.36.

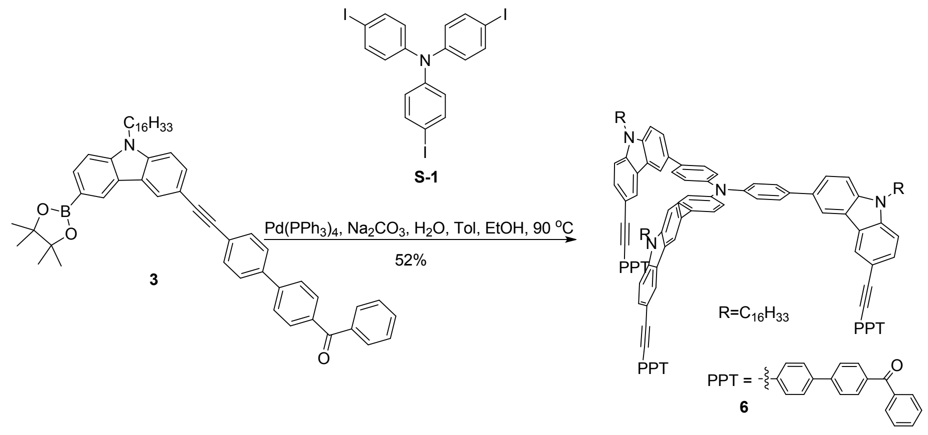

Synthesis of 6: To a Schlenk tube were added **S-1** (47.6 mg, 0.0765 mmol), **3** (220 mg, 0.275 mmol), $Pd(PPh_3)_4$ (8.8 mg, 0.0077 mmol), and Na_2CO_3 (182 mg, 1.72 mmol). Toluene (11.3 mL), water (1.3 mL), and ethanol (1.3 mL) were added into the tube and the mixture was stirred at 90 °C for 18 h. The organic solvents were removed and dichloromethane (30 mL) was added. The solution was washed with water (15 mL) and

brine (15 mL), and the organic layer was dried under Na_2SO_4. The volatiles were removed and the crude product was purified by flash column chromatography (CH_2Cl_2/hexane, 1/3, *V*/*V*) to afford product as a green oil (90 mg, 52%): ^1H NMR (400 MHz, $CDCl_3$) δ 8.39 (d, J = 1.5 Hz, 3H), 8.35 (d, J = 1.7 Hz, 3H), 7.92 – 7.87 (m, 6H), 7.86 – 7.81 (m, 6H), 7.77 (dd, J = 8.5, 1.7 Hz, 3H), 7.73 – 7.58 (m, 30H), 7.54 – 7.45 (m, 9H), 7.38 (t, J = 8.5 Hz, 9H), 4.30 (t, J = 7.3 Hz, 6H), 1.90 (p, J = 7.1 Hz, 6H), 1.26 (d, J = 2.5 Hz, 78H), 0.88 (t, J = 6.7 Hz, 9H). ^{13}C NMR (101 MHz, $CDCl_3$) δ 196.35, 146.54, 144.48, 140.72, 140.21, 139.07, 137.81, 136.44, 132.52, 132.11, 130.90, 130.12, 129.55, 128.44, 128.10, 127.27, 126.86, 125.50, 124.69, 124.34, 124.05, 123.17, 123.11, 118.66, 113.22, 109.36, 109.06, 92.49, 87.56, 43.48, 32.06, 29.85, 29.83, 29.80, 29.76, 29.72, 29.66, 29.55, 29.51, 29.17, 27.45, 22.84, 14.29. MALDI-TOF (*m*/*z*): $[M+H]^+$ calcd. for $C_{165}H_{168}N_4O_3$, 2254.32; found: 2255.92.

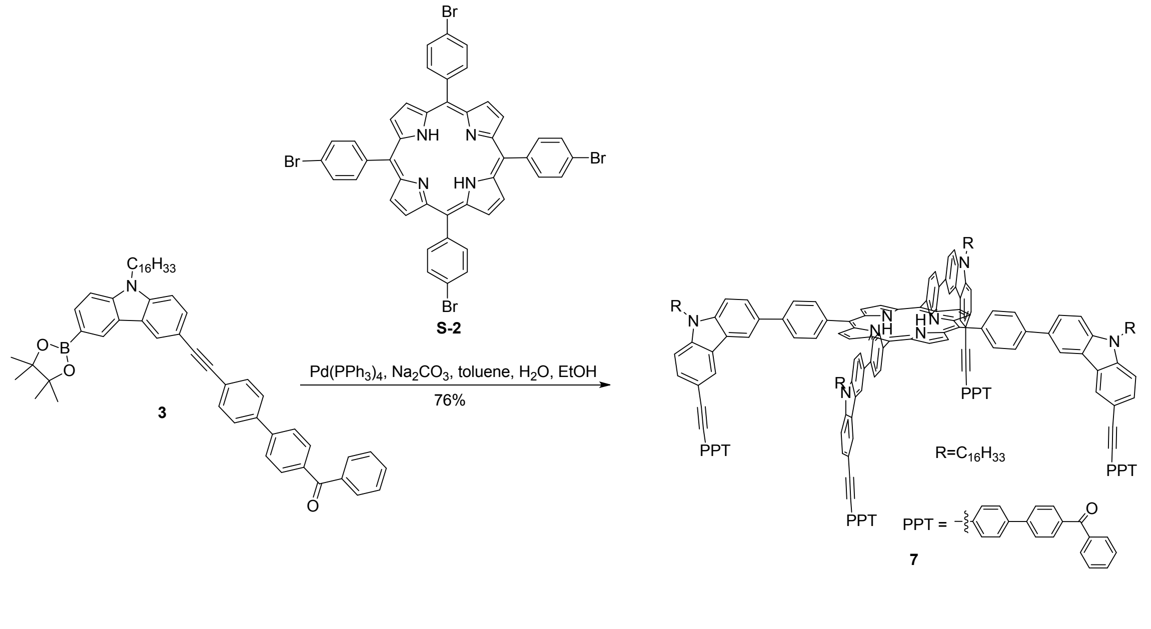

Synthesis of 7: To a Schlenk tube were added **S-2** (53.4 mg, 0.0574 mmol), **3** (220 mg, 0.275 mmol), $Pd(PPh_3)_4$ (8.6 mg, 0.0075 mmol), and Na_2CO_3 (182 mg, 1.72 mmol).

Toluene (15 mL), water (1.7 mL), and ethanol (1.7 mL) were added into the tube and the mixture was stirred at 90 ^{o}C for 18 h. The organic solvents were removed and dichloromethane (40 mL) was added. The solution was washed with water (20 mL) and brine (20 mL), and the organic layer was dried under Na_2SO_4. The volatiles were removed and the crude product was purified by flash column chromatography ($MeOH/CH_2Cl_2$, 1/49, *V*/*V*) to afford product as a purple solid (144 mg, 76%): ^{1}H NMR (400 MHz, $CDCl_3$) δ 9.05 (s, 8H), 8.67 (d, *J* = 1.6 Hz, 4H), 8.51 (d, *J* = 1.5 Hz, 4H), 8.35 (d, *J* = 7.9 Hz, 8H), 8.11 (d, *J* = 7.9 Hz, 8H), 8.07 (dd, *J* = 8.4, 1.7 Hz, 4H), 7.88 (d, *J* = 8.2 Hz, 8H), 7.85 – 7.81 (m, 8H), 7.74 – 7.57 (m, 36H), 7.54 – 7.41 (m, 12H), 4.37 (t, *J* = 7.1 Hz, 8H), 1.96 (p, *J* = 7.1 Hz, 8H), 1.26 (s, 104H), 0.86 (t, *J* = 6.7 Hz, 12H), -2.59 (s, 2H). ^{13}C NMR (101 MHz, $CDCl_3$) δ 196.40, 144.51, 141.15, 140.84, 140.69, 140.62, 139.13, 137.82, 136.46, 135.43, 132.53, 132.14, 130.91, 130.13, 129.69, 128.44, 127.29, 126.88, 126.01, 125.59, 124.47, 124.05, 123.35, 123.28, 120.21, 119.41, 113.44, 109.63, 109.18, 92.45, 87.63, 43.58, 32.07, 29.86, 29.82, 29.79, 29.75, 29.70, 29.58, 29.52, 29.22, 27.50, 22.84, 14.29. MALDI-TOF (*m*/*z*): $[M+H]^+$ calcd. for $C_{240}H_{234}N_8O_4$, 3292.84; found: 3294.61.

General procedures of alkyne metathesis.

Alkyne metathesis using catalyst from L_N: The triphenolamine ligand L_N and the molybdenum precursor were premixed in dry carbon tetrachloride (1.5 mL per 20 mg of molybdenum precursor) and stirred for 20 minutes to generate the catalyst in situ. Then the catalyst solution was added to the monomer solution in CCl_4 and the mixture was stirred at 40 ^{o}C overnight. The reaction was monitored by GPC and NMR. Upon the appearance of a sharp single peak in GPC trace of the reaction mixture, the reaction was

taken out of the glovebox and the precipitates were filtered by vacuum filtration. The filtrate was concentrated and the residue was purified by flash column chromatography.

Alkyne metathesis by using catalyst from L_{Si}: The triphenolsilane ligand L_{Si} and the molybdenum precursor were premixed in dry carbon tetrachloride (1.5 mL per 20 mg of molybdenum precursor) and stirred for 20 minutes to generate the catalyst in situ. Then the catalyst solution was added to the monomer solution in CCl_4 and the mixture was stirred at 55 ^{o}C overnight. The workup procedure is similar to the above one.

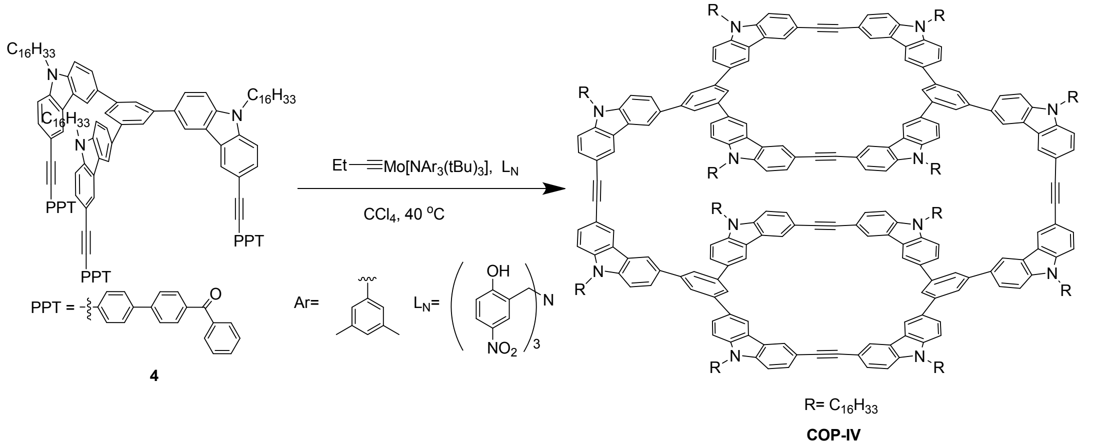

Synthesis of COP-IV: The general procedure described above was followed. The monomer **4** (115 mg, 0.055 mmol), ligand L_N (15 mg, 0.030 mmol), the molybdenum precursor (20 mg, 0.030 mmol) and CCl_4 (6 mL) were used. Crude mixture was purified by flash column chromatography (CH_2Cl_2/hexane, 1/4, *V*/*V*) to provide the pure product as a yellow solid (56 mg, 79%): ^{1}H NMR (500 MHz, $CDCl_3$) δ 8.61 (s, 4H), 8.60 (s, 8H), 8.58(s, 4H),8.41(s, 8H), 8.29(s, 4H), 8.11(s, 8H), 8.08(d, J=10 Hz, 8H), 7.98 (d, J =10 Hz, 4H), 7.74 (d, J = 8.5 Hz, 8H), 7.66 (d, J = 8.2 Hz, 8H), 7.57 (d, J = 8.6 Hz, 4H), 7.49 (d, J = 8.7 Hz, 8H), 7.45 (d, J = 8.7 Hz, 4H), 7.28 (d, 4H), 4.40(s, 8H), 4.09(s, 16H), 1.98(m, 8H), 1.77(m, 16H), 1.27-1.15(m, 348H), 0.90-0.82(m, 87H); ^{13}C NMR (101

MHz, $CDCl_3$) δ 143.0, 142.4, 140.4, 140.3, 133.1, 131.9, 129.7, 128.7, 125.6, 125.2, 124.3, 124.0, 123.3, 123.2, 123.1, 122.8, 119.4, 118.5, 114.2, 114.0, 109.3, 109.1, 108.7, 89.2, 88.0, 43.4, 43.1, 31.9, 29.7, 29.6, 29.5, 29.6, 29.1, 29.0, 27.4, 27.3, 22.7, 14.1. MALDI-TOF (*m/z*): $[M]^+$ calcd. for $C_{372}H_{480}N_{12}$, 5115.79; found: 5115.66.

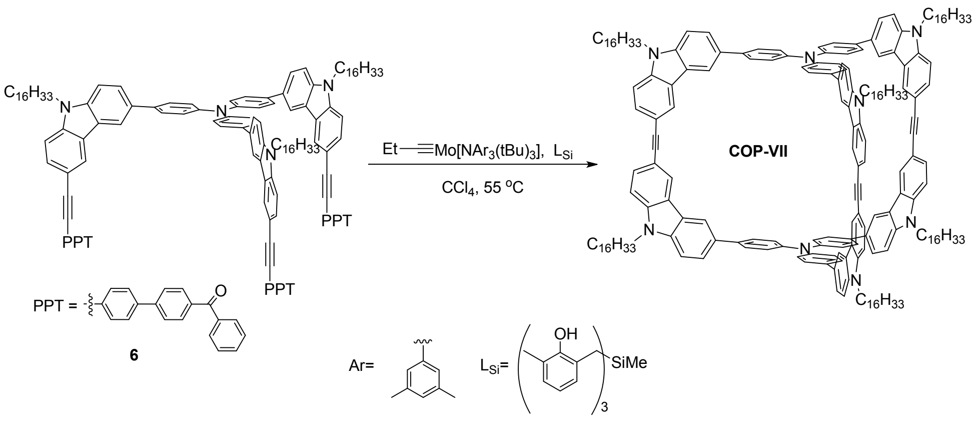

Synthesis of COP-VII: The general procedure described above was followed. The monomer **6** (43 mg, 0.019 mmol), ligand L_{Si} (0.6 mg, 0.0015 mmol), the molybdenum precursor (1.0 mg, 0.0015 mmol) and CCl_4 (0.5 mL) were used. Crude mixture was purified by column chromatography (CH_2Cl_2/hexane, 1/2, *V*/*V*) to provide the pure product as a white solid (23 mg, 84 %): ^{1}H NMR (400 MHz, $CDCl_3$) δ 8.46 (d, J = 1.6 Hz, 6H), 8.37 (s, 6H), 7.74 (d, J = 8.5 Hz, 6H), 7.68 (dd, J = 8.4, 1.6 Hz, 6H), 7.63 (d, J = 8.4 Hz, 12H), 7.45 (d, J = 8.6 Hz, 6H), 7.39 (t, J = 7.5 Hz, 18H), 4.32 (t, J = 6.9 Hz, 12H), 1.90 (dt, J = 14.2, 6.7 Hz, 12H), 1.24 (s, 168H), 0.90 – 0.82 (m, 18H). ^{13}C NMR (101 MHz, $CDCl_3$) δ 140.32, 140.22, 136.79, 132.77, 129.11, 128.42, 125.67, 125.47, 124.78, 124.04, 123.22, 118.86, 114.20, 109.22, 108.94, 89.13, 43.51, 32.07, 30.46, 29.85, 29.83, 29.80, 29.76, 29.73, 29.67, 29.56, 29.51, 29.21, 27.49, 22.84, 14.29. MALDI-TOF (*m/z*): $[M+H]^+$ calcd. for $C_{210}H_{258}N_8$, 2893.05; found: 2895.13.

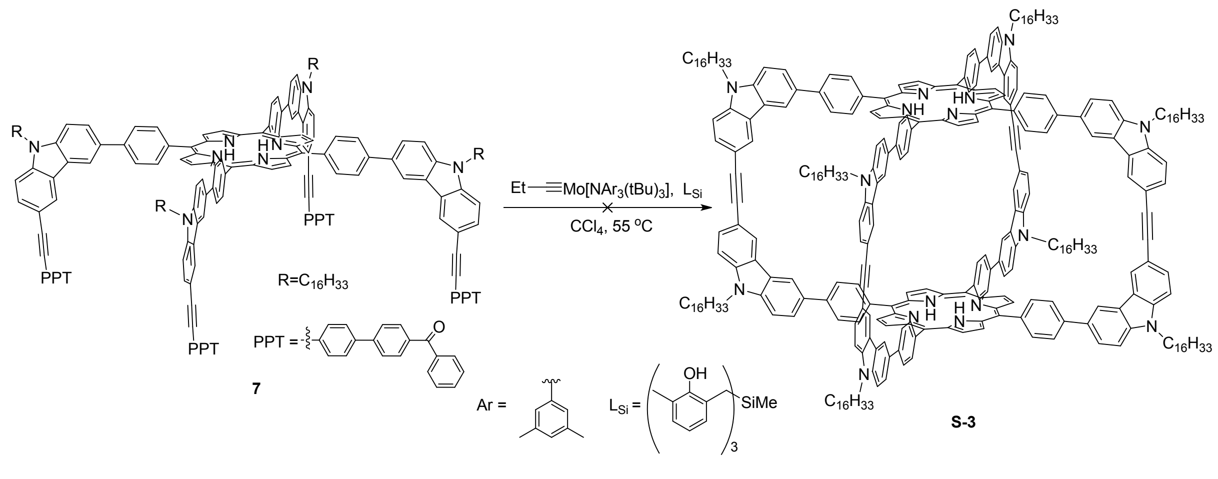

Attempted synthesis of S-3: The general procedure described above was followed. The monomer **7** (5.6 mg, 0.0017 mmol), ligand L_{Si} (0.2 mg, 0.0005 mmol), the molybdenum precursor (0.3 mg, 0.0005 mmol) and CCl_4 (0.5 mL) were used. Lots of precipitates were formed during the reaction. After stirring overnight (~16 h) at 55 ^{o}C, the reaction was cooled to rt. We did not observe any noticeable amount of compound **7** in the ^{1}H NMR spectrum of the crude reaction mixture, indicating all the starting materials are consumed. The precipitates were filtered and the filtrate was concentrated. The ^{1}H NMR spectrum of the residue (trace amount) obtained from the filtrate only shows PPT-≡-PPT, byproduct of the metathesis reaction. We also conducted IR absorption and MALDI-TOF mass analysis on the precipitates. We could not obtain any insightful information from the IR spectra of the precipitates. MALDI-TOF spectra shows the molecular ion peaks corresponding to a dimer ($C_{320}H_{364}N_{16}$, *m*/*z*: $[M+Li]^+$ calcd. for, 4437.83; found: 4439.09) and a tetramer ($C_{640}H_{728}N_{32}$, *m*/*z*: $[M+Li]^+$ calcd. for, 8868.81; found: 8872.66). However, further determination of their structures was difficult due to their low solubility and limited amount mixed in the precipitates. Most of the precipitates are likely insoluble oligomers or polymers.

Kinetic study of COP formation

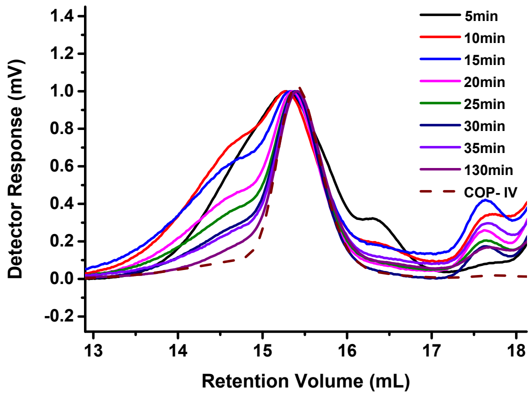

Figure S1. GPC traces of reaction mixtures and **COP-IV**.

Computational method

The Amber 11.0 molecular dynamics program package[4] was used to optimize the structures of cages and cage-fullerene complexes by energy minimization for 1000 steps. The force field used for the cages and fullerenes was the general Amber force field (GAFF)[5] with the charge parameters computed by the AM1-BCC method.

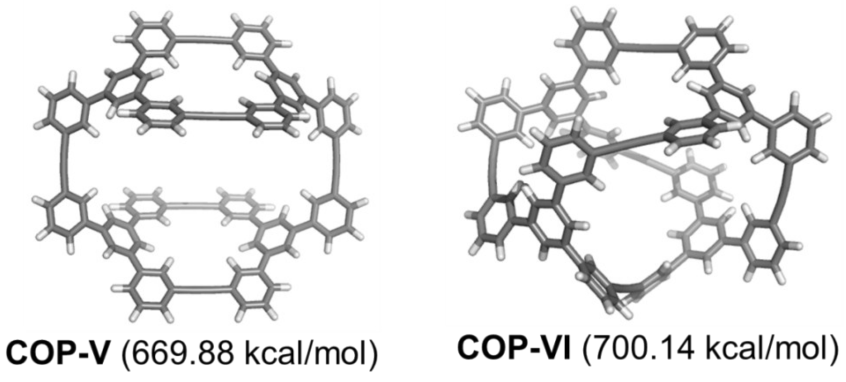

Figure S2. Energy minimized structures of **COP-V** and **COP-VI** and their corresponding energies.

NMR characterization of COP-IV

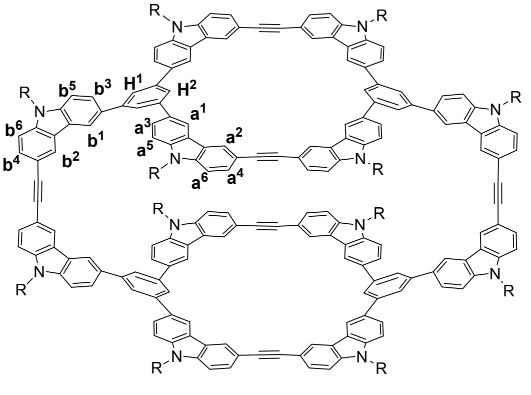

COP-III (R = $C_{16}H_{33}$)

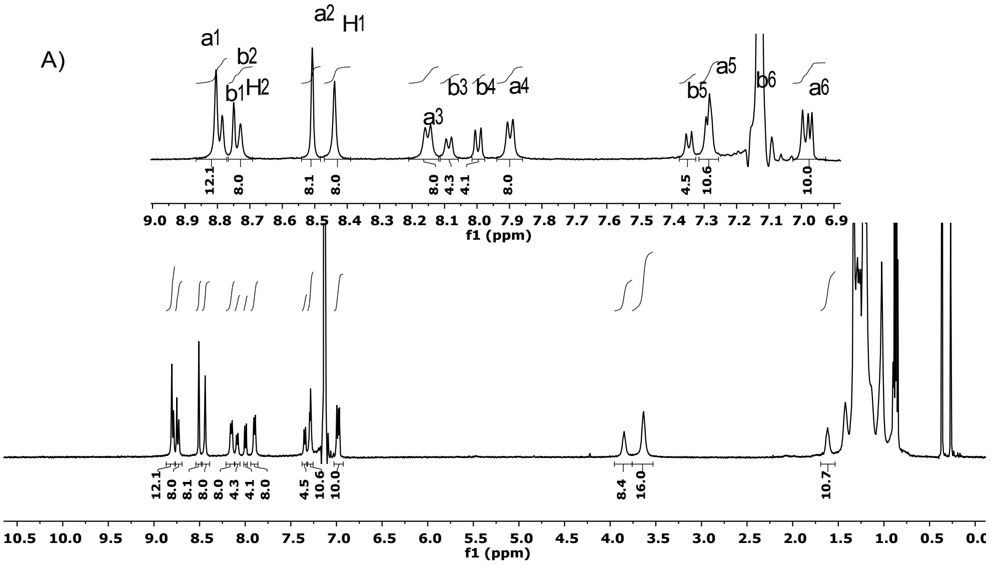

B)

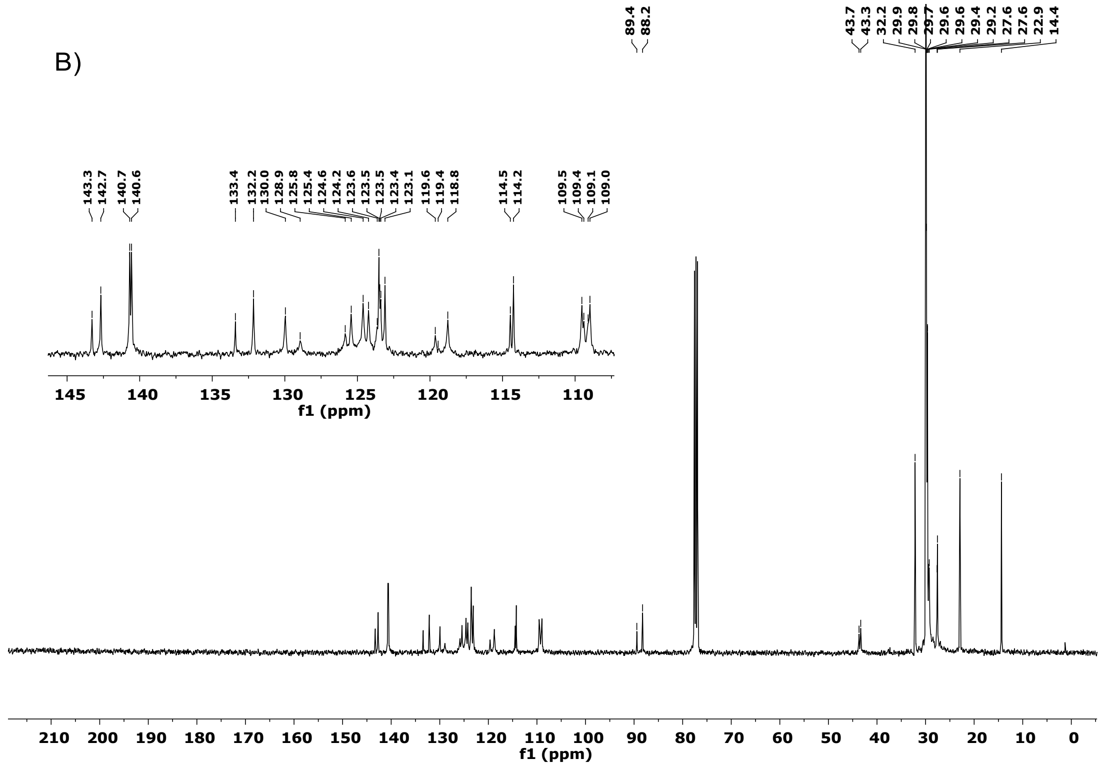

Figure S3. ^{1}H NMR spectrum of **COP-IV** in C_6D_6 (A) and ^{13}C NMR spectrum in $CDCl_3$ (B).

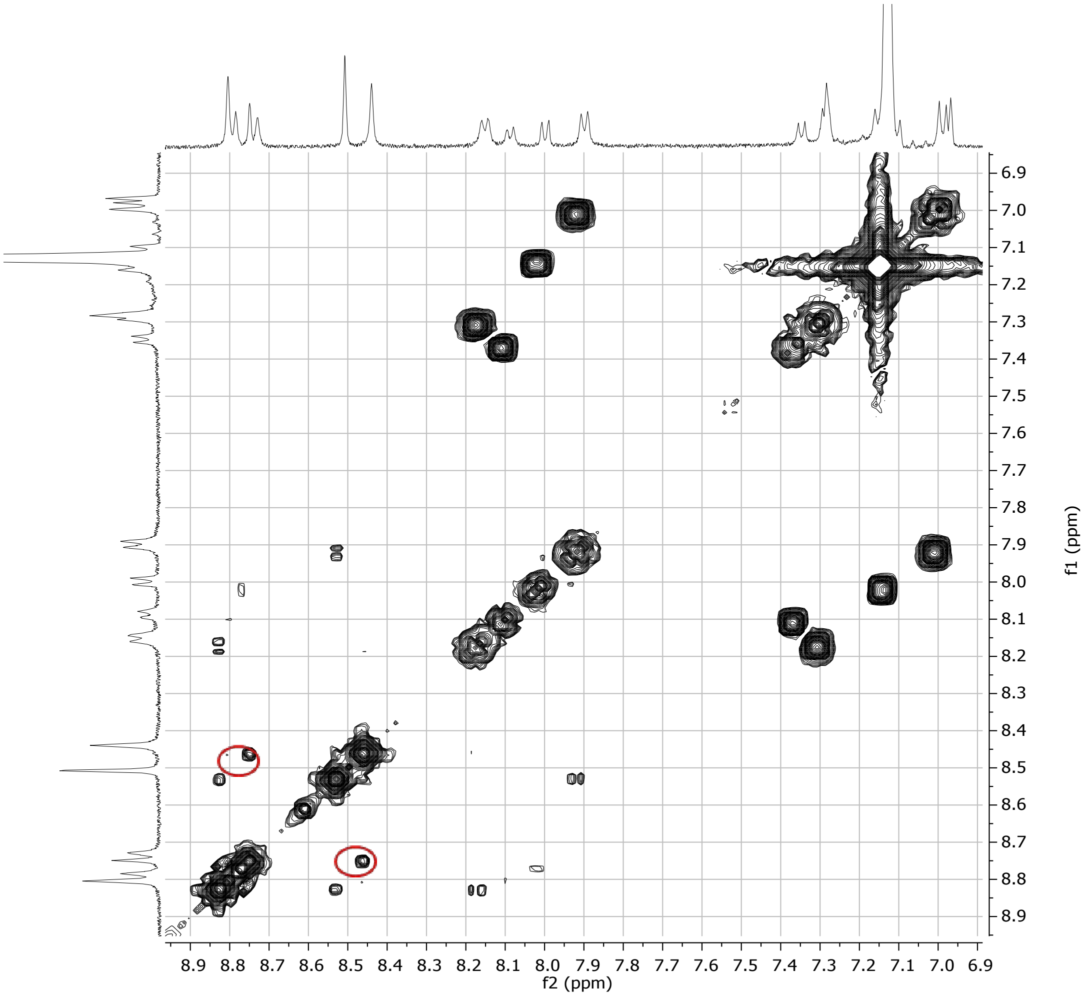

Figure S4. gCOSY spectrum of **COP-IV** in C_6D_6. The labeled cross peaks belonging to center benzene protons proves that the two sets of peaks are from one molecule instead of two different species.

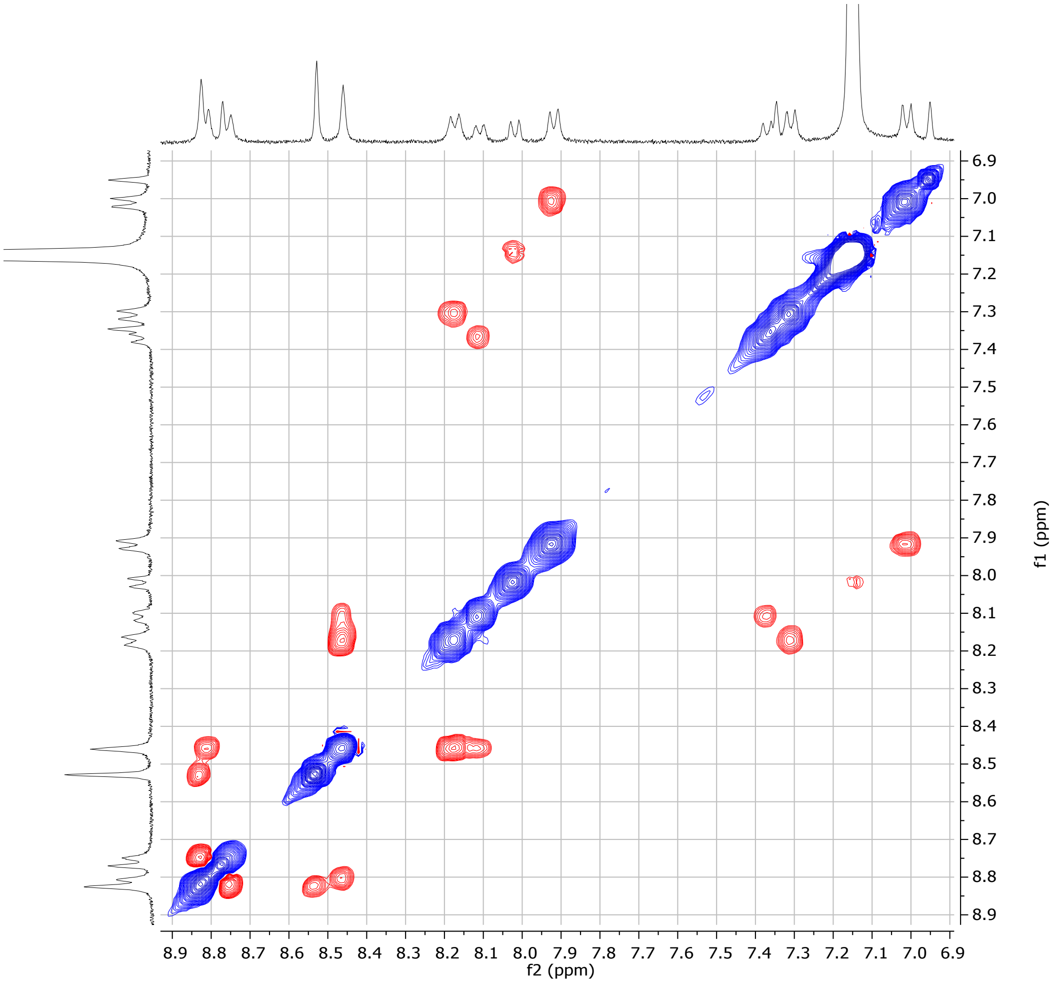

Figure S5. ROESY spectrum of **COP-IV** in C_6D_6.

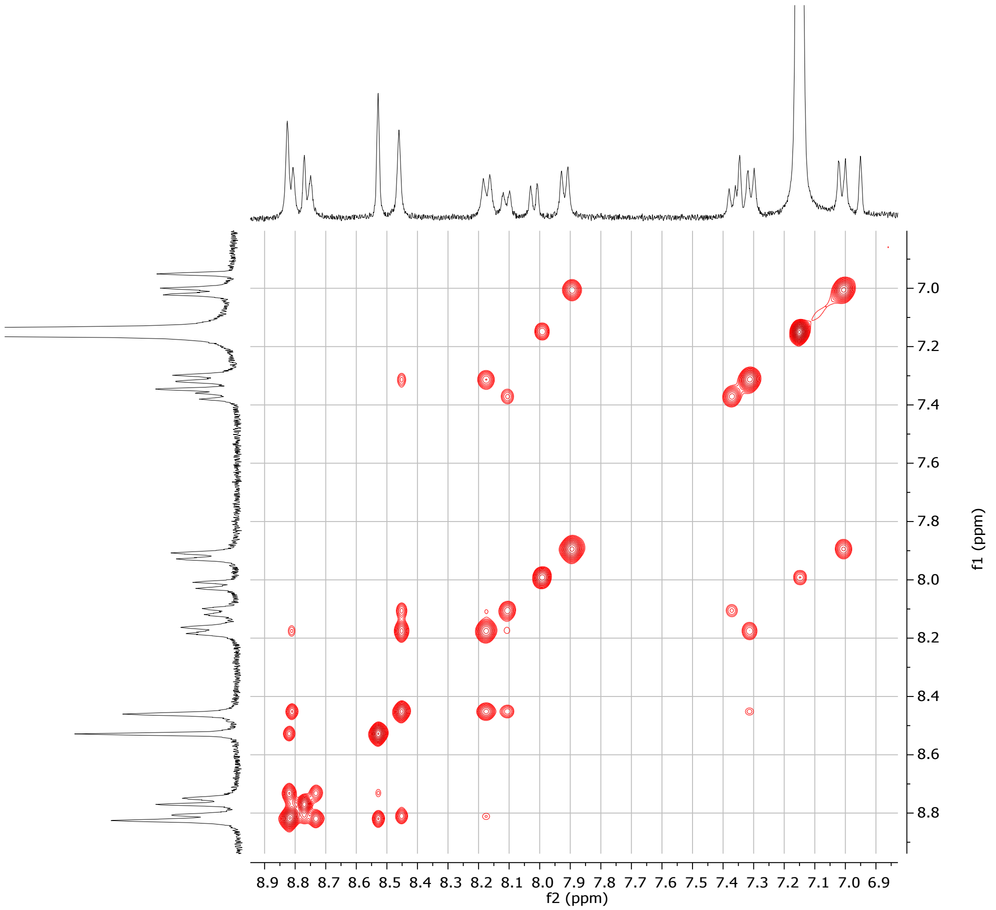

Figure S6. NOESY spectrum of **COP-IV** at room temperature in C_6D_6.

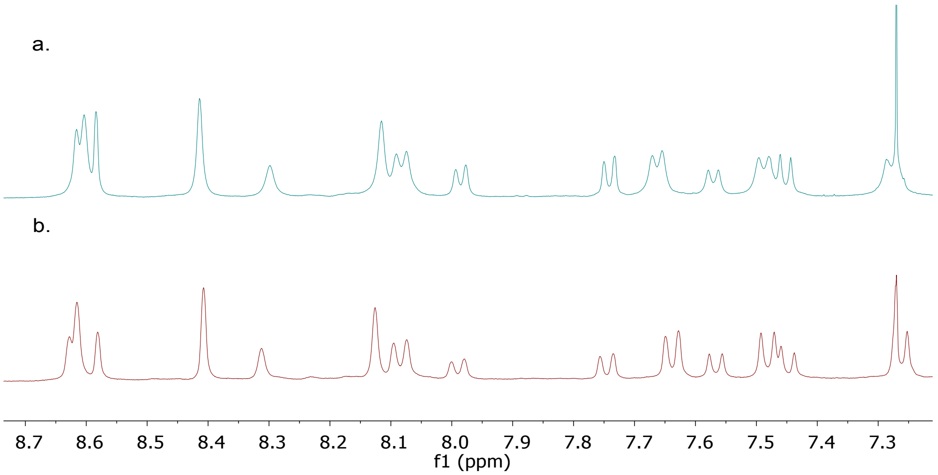

Figure S7. Variant-temperature ^{1}H NMR spectra of **COP-IV** at 20 ^{o}C (a) and 59 ^{o}C (b) in $CDCl_3$.

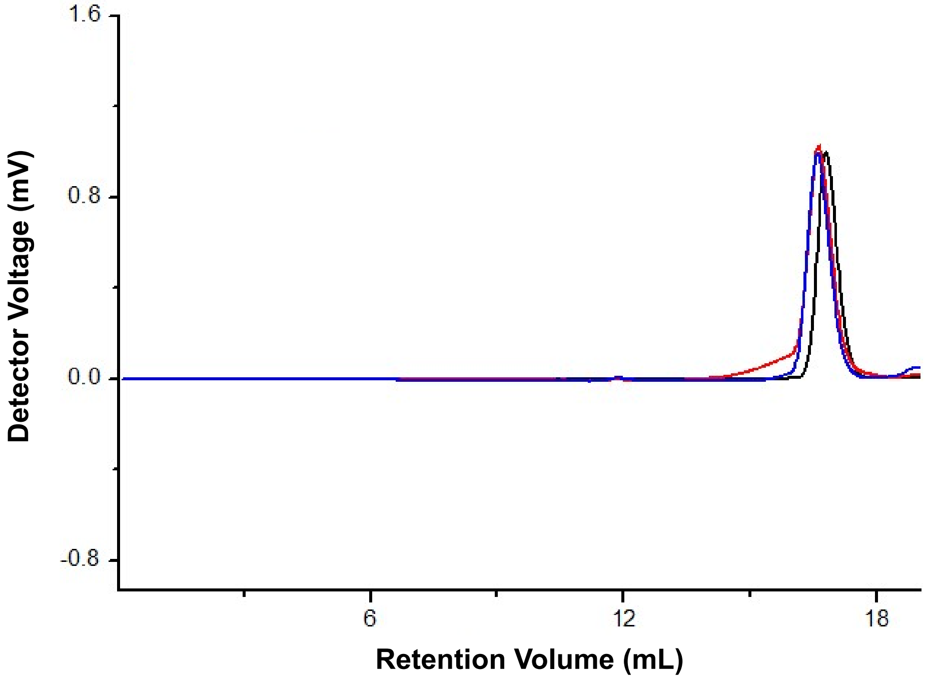

Figure S8. Normalized GPC traces of monomer **4** (black), crude reaction mixture after workup (red), and pure **COP-IV** (blue).

NMR spectra of new compounds

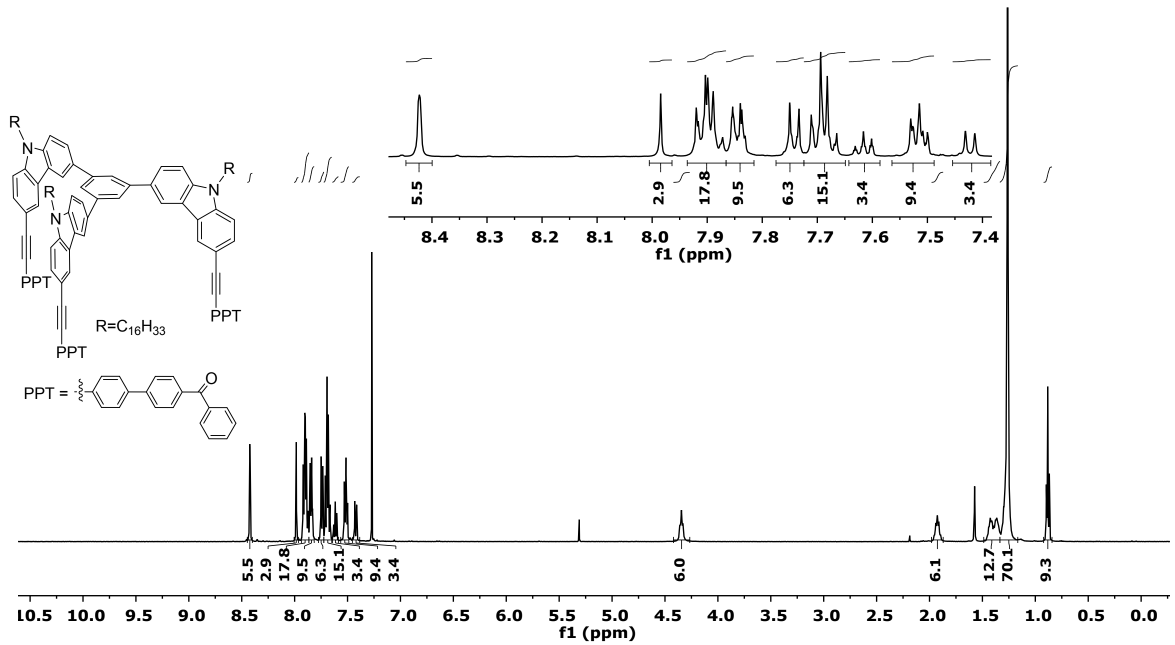

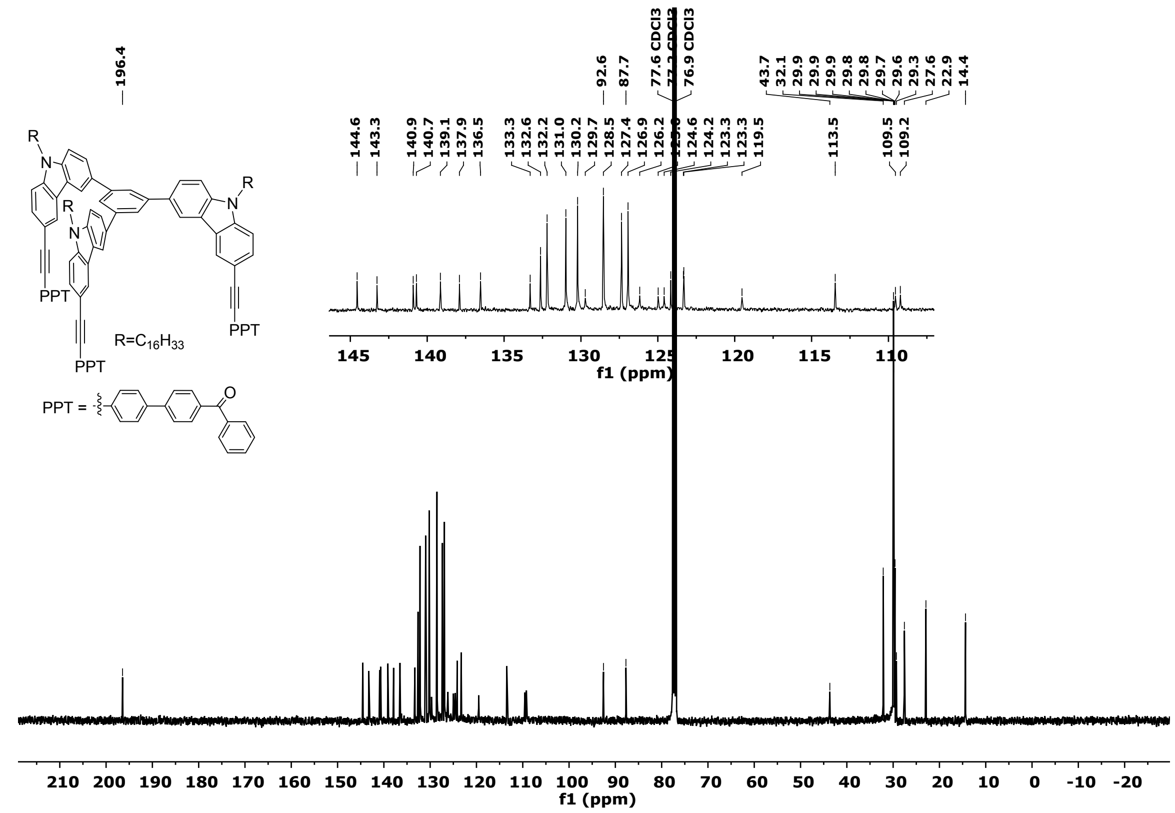

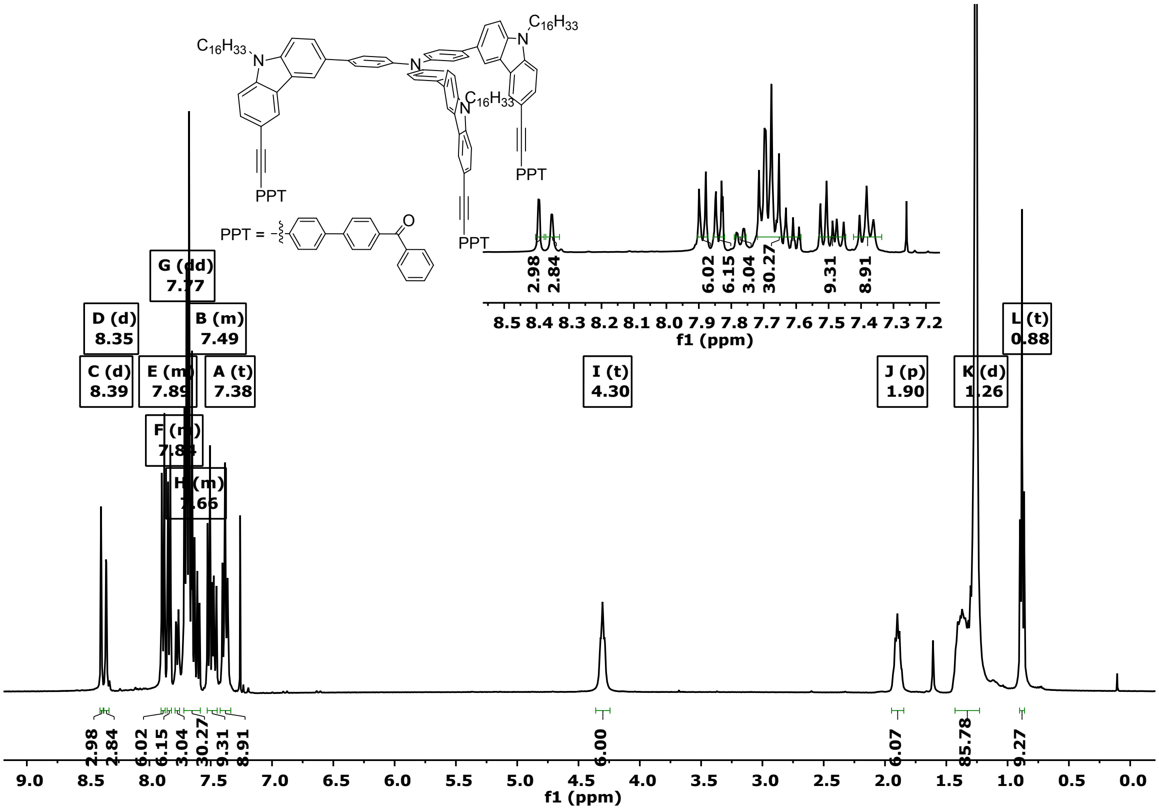
C₁₆H₃₃
PPT
PPT =
G (dd)
7.77
D (d)
8.35
B (m)
7.49
C (d)
8.39
E (m)
7.89
A (t)
7.38
I (t)
4.30
J (p)
1.90
K (d)
1.26
L (t)
0.88
H (m)
7.66
f1 (ppm)

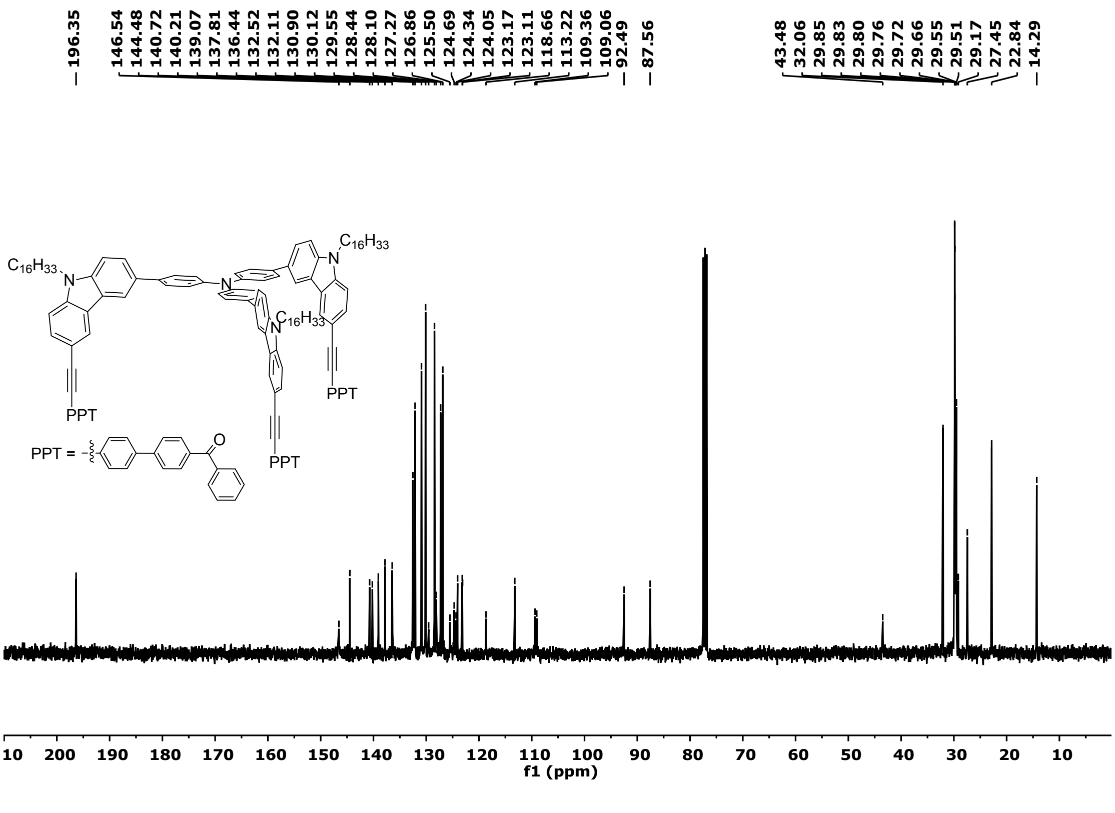
196.35
146.54
144.48
140.72
140.21
139.07
137.81
136.44
132.52
132.11
130.90
130.12
129.55
128.44
128.10
127.27
126.86
125.50
124.69
124.34
124.05
123.17
123.11
118.66
113.22
109.36
109.06
92.49
87.56
43.48
32.06
29.85
29.83
29.80
29.76
29.72
29.66
29.55
29.51
29.17
27.45
22.84
14.29
PPT =
PPT
f1 (ppm)

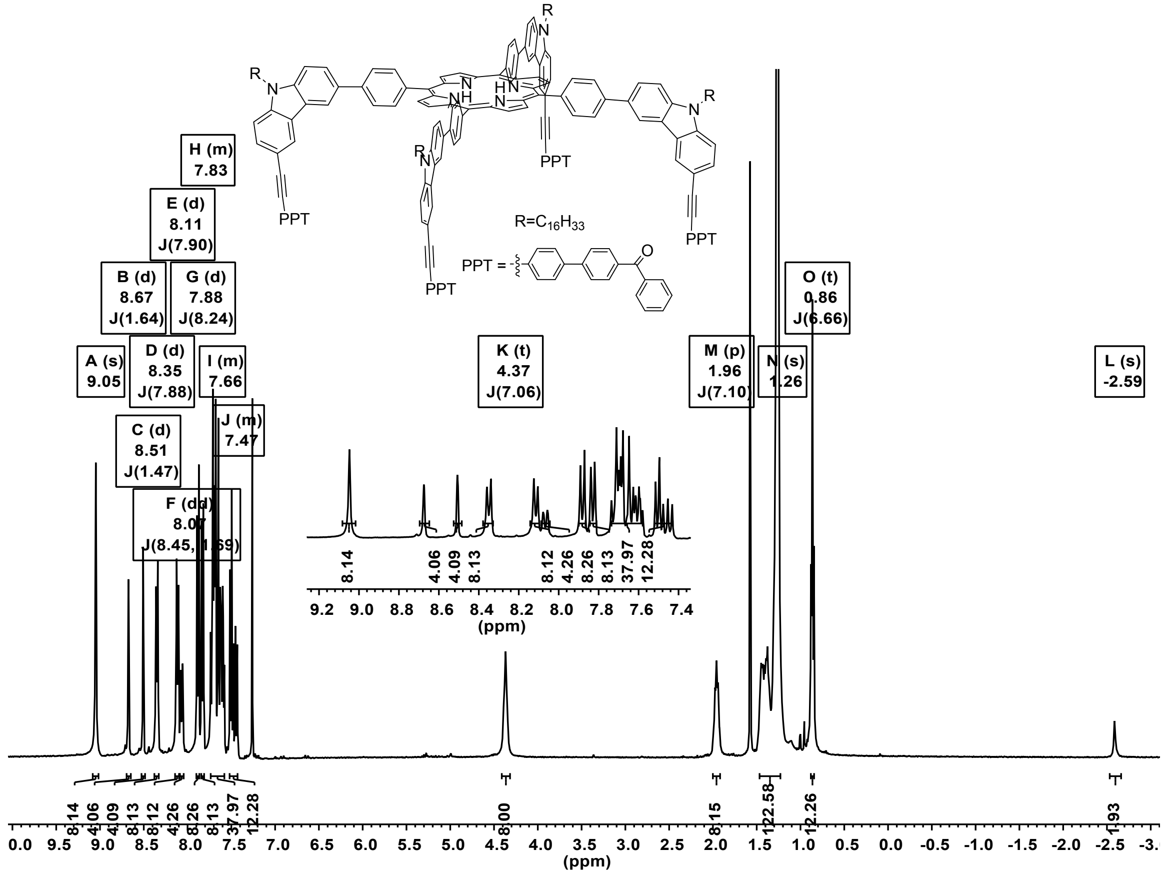

A (s) 9.05
B (d) 8.67 J(1.64)
C (d) 8.51 J(1.47)
D (d) 8.35 J(7.88)
E (d) 8.11 J(7.90)
F (dd) 8.07 J(8.45, 1.69)
G (d) 7.88 J(8.24)
H (m) 7.83
I (m) 7.66
J (m) 7.47
K (t) 4.37 J(7.06)
M (p) 1.96 J(7.10)
N (s) 1.26
O (t) 0.86 J(6.66)
L (s) -2.59
R=C16H33
PPT =
PPT
(ppm)

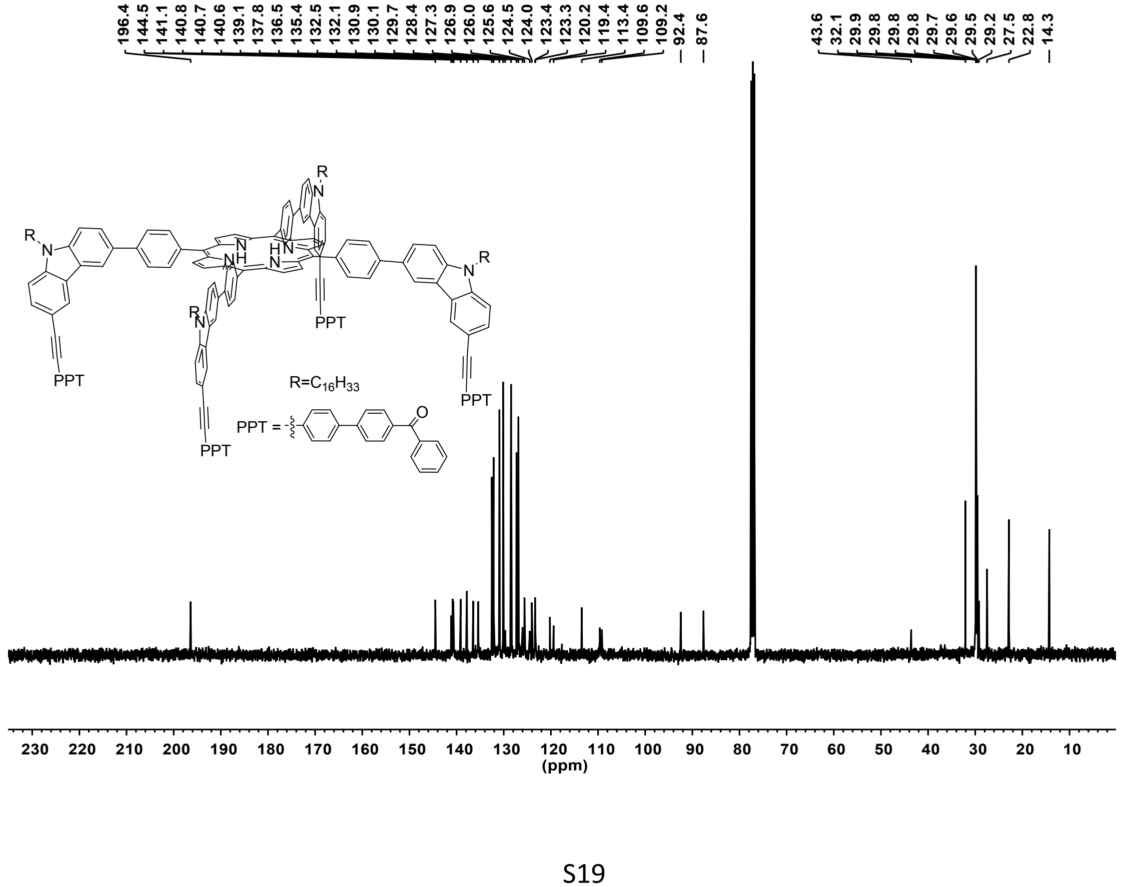

196.4 144.5 141.1 140.8 140.7 140.6 139.1 137.8 136.5 135.4 132.5 132.1 130.9 130.1 129.7 128.4 127.3 126.9 126.0 125.6 124.5 124.0 123.4 123.3 120.2 119.4 113.4 109.6 109.2 92.4 87.6
43.6 32.1 29.9 29.8 29.8 29.8 29.7 29.6 29.5 29.2 27.5 22.8 14.3
R=C16H33
PPT =
PPT
(ppm)

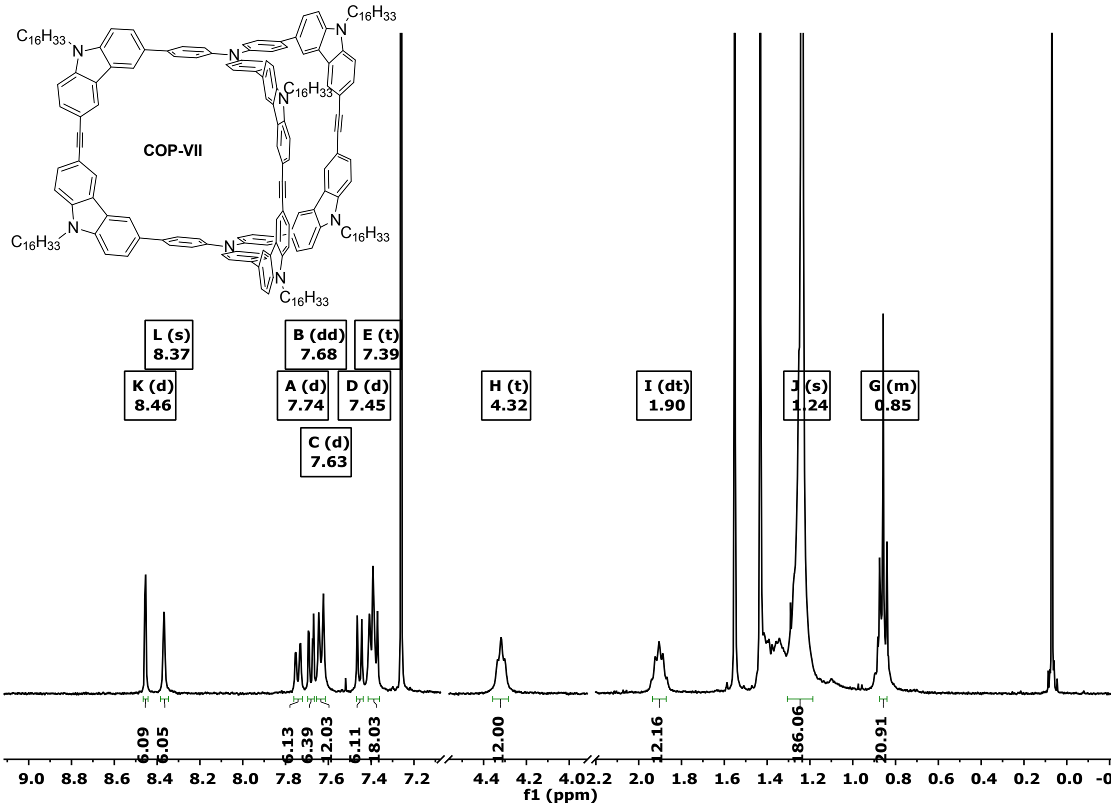
C16H33
COP-VII
L (s) 8.37
B (dd) 7.68
E (t) 7.39
K (d) 8.46
A (d) 7.74
D (d) 7.45
C (d) 7.63
H (t) 4.32
I (dt) 1.90
J (s) 1.24
G (m) 0.85
6.09
6.05
6.13
6.39
12.03
6.11
18.03
12.00
12.16
186.06
20.91
9.0 8.8 8.6 8.4 8.2 8.0 7.8 7.6 7.4 7.2 4.4 4.2 4.0 2.2 2.0 1.8 1.6 1.4 1.2 1.0 0.8 0.6 0.4 0.2 0.0
f1 (ppm)

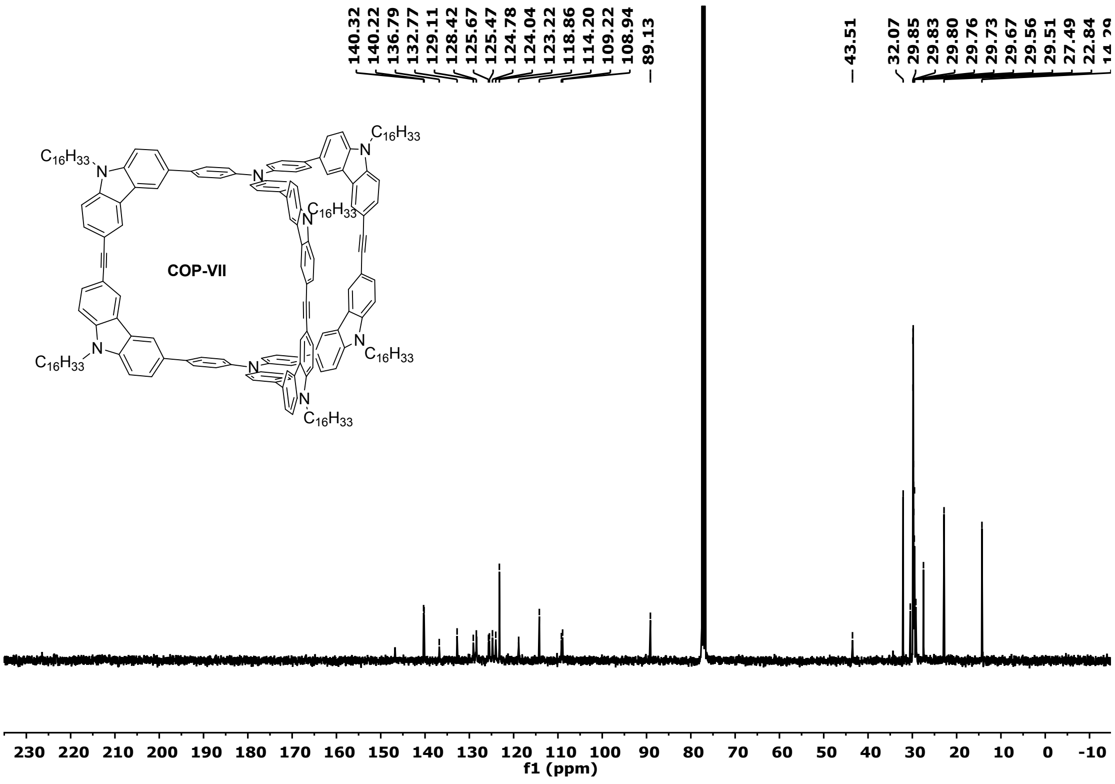
140.32
140.22
136.79
132.77
129.11
128.42
125.67
125.47
124.78
124.04
123.22
118.86
114.20
109.22
108.94
89.13
43.51
32.07
29.85
29.83
29.80
29.76
29.73
29.67
29.56
29.51
27.49
22.84
C16H33
COP-VII
230 220 210 200 190 180 170 160 150 140 130 120 110 100 90 80 70 60 50 40 30 20 10 0 -10
f1 (ppm)

References

1. Q. Wang, C. Yu, H. Long, Y. Du, Y. Jin and W. Zhang, *Angew. Chem. Int. Ed.*, 2015, **54**, 7550-7554.
2. J. Feng, C. Zhang, Y. Li and M. Yang, *J. Appl. Polym. Sci.*, 2011, **121**, 217-225.
3. X. Liu, Y. Xu, Z. Guo, A. Nagai and D. Jiang, *Chem. Commun.*, 2013, **49**, 3233-3235.
4. D.A. Case, T.A. Darden, I. T.E. Cheatham, C.L. Simmerling, J. Wang, R.E. Duke, R. Luo, R.C. Walker, W. Zhang, K.M. Merz, B. Roberts, B. Wang, S. Hayik, A. Roitberg, G. Seabra, I. Kolossváry, K.F. Wong, F. Paesani, J. Vanicek, J. Liu, X. Wu, S.R. Brozell, T. Steinbrecher, H. Gohlke, Q. Cai, X. Ye, J. Wang, M.-J. Hsieh, G. Cui, D.R. Roe, D.H. Mathews, M.G. Seetin, C. Sagui, V. Babin, T. Luchko, S. Gusarov, A. Kovalenko and P. A. Kollman, *AMBER 11*, University of California, San Francisco, 2010.
5. J. M. Wang, R. M. Wolf, J. W. Caldwell, P. A. Kollman and D. A. Case, *J. Comput. Chem.*, 2004, **25**, 1157-1174.
